# Genomic diversity in *Fructobacillus* spp. isolated from fructose-rich niches

**DOI:** 10.1371/journal.pone.0281839

**Published:** 2023-02-16

**Authors:** Florencia Mohamed, Luciana G. Ruiz Rodriguez, Azul Zorzoli, Helge C. Dorfmueller, Raúl R. Raya, Fernanda Mozzi

**Affiliations:** 1 Centro de Referencia para Lactobacilos (CERELA)-CONICET, San Miguel de Tucumán, Tucumán, Argentina; 2 Division of Molecular Microbiology, School of Life Sciences, University of Dundee, Dundee, United Kingdom; University of Torino, ITALY

## Abstract

The *Fructobacillus* genus is a group of obligately fructophilic lactic acid bacteria (FLAB) that requires the use of fructose or another electron acceptor for their growth. In this work, we performed a comparative genomic analysis within the genus *Fructobacillus* by using 24 available genomes to evaluate genomic and metabolic differences among these organisms. In the genome of these strains, which varies between 1.15- and 1.75-Mbp, nineteen intact prophage regions, and seven complete CRISPR-Cas type II systems were found. Phylogenetic analyses located the studied genomes in two different clades. A pangenome analysis and a functional classification of their genes revealed that genomes of the first clade presented fewer genes involved in the synthesis of amino acids and other nitrogen compounds. Moreover, the presence of genes strictly related to the use of fructose and electron acceptors was variable within the genus, although these variations were not always related to the phylogeny.

## Introduction

The genus *Fructobacillus* is a group of rod-shaped heterofermentative lactic acid bacteria (LAB) that was described just over a decade ago by Endo and Okada [1]. Initially, this genus was included in the *Leuconostocaceae* family but in 2020, due to its phylogenetic position and the morphological and biochemical characteristics of its members, it was placed into the *Lactobacillaceae* family [2, 3]. Up to date, the genus *Fructobacillus* is composed of eleven species: *F*. *durionis*, *F*. *fructosus* (type species), *F*. *ficulneus*, *F*. *pseudoficulneus*, *F*. *tropaeoli*, *F*. *papyriferae*, *F*. *papyrifericola*, *F*. *broussonetiae*, *F*. *parabroussonetiae*, *F*. *cardui* and *F*. *apis*. The *Fructobacillus* species are classified as obligatory fructophilic lactic acid bacteria (FLAB) [4], as a result of their preference for D-fructose over D-glucose as growth substrate, related to their requirement for an electron acceptor (oxygen, pyruvate, or fructose) during glucose dissimilation. All members of this genus produce equimolar amounts of lactic acid and acetic acid and a small amount of ethanol as main end-products [2, 4, 5]. The *Fructobacillus* species only ferment a limited number of carbohydrates, mainly D-glucose and D-fructose; and some species are known as osmotolerant [1, 4, 6]. These organisms have been found in flowers, fruits, and insects associated with these environments or related fermented products; all niches linked to high fructose content [7–14].

Due to their unique features, and dominance and adaptation to specific environments, some *Fructobacillus* species have been studied to assess their technological potential. Since *Fructobacillus* and other FLAB organisms have been found in environments associated with bees (high fructose consumer insects) [8, 10, 12, 15–17], several studies have reported the potential of *Fructobacillus* and its by-products to improve the health of honey bees [12, 18]. These findings are highly relevant given that bees are pollinators *par excellence* in nature, and they are declining worldwide. In addition, the genus *Fructobacillus* deserves a marked interest for its potential application in the food industry. The relevance of some species in spontaneous food fermentation was evidenced in some processes due to their dominance, such as in Tempoyak and cocoa bean fermentation [19–21]. *Fructobacillus* organisms are able to metabolize fructose preferentially and colonize unusual niches, features of great interest for their exploitation in food fermentation [22]. A reduction of fructose content in food is desirable since a high intake of this sugar contributes to multiple health consequences, such as insulin resistance, obesity, liver disorders, diabetes and high blood pressure [23, 24]. *Fructobacillus* organisms can consume fructose by two pathways: i) as energy substrate (through the phosphoketolase pathway), producing lactic and acetic acids as main fermentation products, and ii) as electron acceptor, reducing this sugar to mannitol [22, 25]. Mannitol is a naturally occurring polyol that is mainly employed as a low-calorie sweetener in food manufacturing. Due to its zero glycemic and insulinemic index, it is suitable as food constituent in people suffering diabetes [26]. Moreover, it also contributes to increase shelf-life of food by reducing the crystallization tendency of sugars [27]. In this regard, *Fructobacillus* organisms are efficient mannitol producers, as previously observed in recent studies [28–30]. High amounts (82 g/L) of high-quality mannitol from fructose were obtained under optimized conditions with *F*. *tropaeoli* CRL 2034 [29]. Conversion efficiency of mannitol by this strain is one of the highest reported for a LAB strain up to date [22, 31, 32].

FLAB are able to extend the shelf life and increase the antioxidant level of food [4]. Acetic acid, mandatorily produced by *Fructobacillus* organisms to counteract the deficiency in ethanol synthesis, exhibits inhibitory effects against certain food spoilage-associated microorganisms [33–36]. FLAB are also capable of modifying plant secondary metabolites during plant fermentation, enhancing the functional and nutritional properties of plant-based products [22]. For instance, *F*. *fructosus* strains have been shown to convert p-coumaric and caffeic acid to phenolic acid derivatives with higher biological activities than their precursors [15]. Additionally, some *Fructobacillus* strains can reduce the fermentable oligosaccharides, disaccharides, monosaccharides, and polyols (FODMAPs) present in food, reducing the risk of the onset of irritable bowel syndrome (IBS) symptoms and other functional gut disorders [37].

Advances in sequencing technologies and the constant development of new or more powerful bioinformatics tools have led to a breakthrough in genomic studies. In this context, the number of available microbial genomes is constantly increasing. Due to the relatively new characterization of the genus *Fructobacillus*, a limited number of genomes have been described. Only a few genomic studies on *Fructobacillus* genomes have been published but they have been enough to reveal that these bacteria have adapted to their specific niches through reductive evolution [25, 38]. Endo et al. [38] performed a comparative genomic analysis between the draft genomes of five *Fructobacillus* spp. and nine *Leuconostoc* spp strains. The results showed that *Fructobacillus* spp. had a smaller genome size (1.49 ± 0.30 Mbp), higher G+C content (≈ 44%) and fewer protein-coding sequences (CDSs) than *Leuconostoc* spp. Furthermore, these authors concluded that *Fructobacillus* showed a reduction in the number of genes involved in carbohydrate transport and metabolism (5.1% in *Fructobacillus* vs 8.8% in *Leuconostoc*) as well as the number of genes related to energy production and conversion, suggesting the existence of simpler energy systems. These genomic analyses showed that *Fructobacillus* strains possess none or at most one gene for the phosphotransferase system (PTS), which is a major transport system in LAB. This niche-specific reductive adaptation, also observed in other FLAB such as *Apilactobacillus kunkeei*, *A*. *apinorum*, and *Fructilactobacillus florum* [39–41] would be a way to simplify cell metabolism considering nutrient availability in fructose-rich niches [22]. In addition, *Fructobacillus* spp. were reported to be the first heterofermentative LAB to lack the *adhE* gene, which encodes a bifunctional alcohol–acetaldehyde dehydrogenase [38]. The absence of *adhE* does not allow this genus to regenerate NAD^+^ by converting acetyl-CoA to ethanol. Therefore, NAD^+^ is regenerated through the conversion of fructose to mannitol, step catalyzed by the mannitol 2-dehydrogenase enzyme (MDH) [2].

Although the genomic properties of some *Fructobacillus* spp. were compared to closely related genus genomes [38], differences within the *Fructobacillus* genus have not been studied in detail yet. In addition, genomes of *F*. *papyriferae*, *F*. *papyrifericola*, *F*. *broussonetiae* and *F*. *parabroussonetiae*, *F*. *cardui and F*. *apis* were not previously used in a comparative analysis. In this study, we deepened the knowledge on the bacterial metabolism of all *Fructobacillus* members from a genomic viewpoint, which may provide relevant information of their biotechnological potential. Thus, a comparative genomic analysis of the genus *Fructobacillus* using all available genomes to the time of writing this article was performed to investigate its pangenome, characterize its mobilome, and compare some metabolic pathways within the group.

## Materials and methods

### Bacterial genomes and DNA extraction

In this study, all available *Fructobacillus* genomes to date (December 2022) were used. Twenty-two available online sequences were retrieved from the National Center for Biotechnology Information (NCBI) database. Genome GenBank accession numbers are listed in Table 1. The draft genome of the mannitol-producer *F*. *tropaeoli* CRL 2034, previously sequenced and characterized by our team [42], was included in this comparative analysis. Furthermore, the genome of the strain *Fructobacillus* sp. CRL 2054, isolated from ripe fig fruit (26.8241405 S 65.2226028 W) in Tucumán, Argentina [30], was sequenced and included in this study.

**Table 1:** Genomic features of *Fructobacillus* genomes used in this study.

Strain	GenBank no.	Genome size (Mb)	G+C content (%)	No. of CDS^a^	Isolation source	Contigs	Scaffolds	N50	L50	Completeness (%)^b^	Contamination (%)^b^	Clade^c^
*Fructobacillus sp*. CRL 2054	JACOFN000000000	1.33	44.4	1321	Fig	28	28	101455	5	98.63	1.09	1
*F*. *durionis* DSM 19113^T^	FOLI00000000	1.33	44.8	1255	Fermented durian	17	17	175703	2	99.18	1.09	1
*F*. *fructosus* KCTC 3544^T^	JQBH00000000	1.48	44.2	1486	Flower	45	45	77208	7	98.63	0.00	1
*F*. *fructosus* DPC 7238	JACTNH000000000	1.38	44.7	1388	Flower	28	28	155038	3	98.63	0.00	1
*F*. *ficulneus* JCM 12225^T^	BBXQ00000000	1.54	43.9	1427	Fig	179	28	19924	22	97.54	2.05	2
*F*. *pseudoficulneus* DSM 15468^T^	FNWS00000000	1.41	44.5	1323	Fig	18	18	284129	2	98.91	0.55	2
*F*. *sp*. EFB-N1	LDUY00000000	1.64	43.7	1629	Honeybee	68	68	56920	10	98.63	0.00	2
*F*. *tropaeoli* F214-1^T^	BBXT00000000	1.69	44.2	1625	Flower	122	101	84243	7	98.63	0.00	2
*F*. *tropaeoli* CRL 2034	WNLV00000000	1.66	44.6	1514	Fig	20	20	1007334	1	98.63	0.00	2
*F*. *tropaeoli* RD012353	BOJU00000000	1.75	44.0	1641	Flower	18	18	280510	3	98.63	0.00	2
*F*. *papyriferae* M1-10^T^	JAAMFI000000000	1.26	46.3	1223	Paper mulberry	7	6	753209	1	98.63	0.00	1
*F*. *papyriferae* M1-13	JAJNCC000000000	1.26	46.3	1223	Paper mulberry	8	8	318564	2	98.63	0.00	1
*F*. *papyrifericola* M1-21^T^	JAAMFJ000000000	1.30	48.5	1269	Paper mulberry	9	9	731438	1	98.63	1.09	1
*F*. *broussonetiae* M2-14^T^	JAAMFK000000000	1.26	46.4	1249	Paper mulberry	13	13	185189	3	98.63	0.18	1
*F*. *parabroussonetiae* S1-1^T^	JAAMFL000000000	1.22	46.5	1184	Paper mulberry	14	14	137716	4	98.63	0.00	1
*F*. *cardui* M131^T^	JAJIAL000000000	1.56	43.76	1647	Flower	41	41	137862	4	98.09	0.00	2
*Fructobacillus* sp. KI3_B9	CP097122	1.41	43.64	1339	Oriental Cockroach	1	1	1414204	1	98.09	0.00	2
*Fructobacillus* sp. M158	JAJIAM000000000	1.30	46.19	1289	Flower	18	18	204722	2	98.09	0.00	1
*F*. *apis* W13^T^	JAMWYK000000000	1.29	48.29	1224	Honeybee	13	13	322238	2	98.09	0.18	1
*F*. *fructosus* strain 13	JAJIAU000000000	1.31	44.89	1281	Honey	30	30	107090	5	98.63	0.00	1
*F*. *fructosus* MAG1 ^d^	CAMKYK000000000	1.34	44.9	1330	Honeybee	8	8	263347	2	98.63	0.00	1
*F*. *fructosus* MAG2 ^d^	CAMKVY000000000	1.34	45.02	1352	Honeybee	16	16	131058	4	98.09	0.00	1
*F*. *fructosus* MAG3 ^d^	CAMLOM000000000	1.15	45.63	1138	Honeybee	47	47	37723	9	97.27	0.27	1
*Fructobacillus* sp. MAG4 ^d^	CAMKWM000000000	1.55	44.02	1458	Honeybee	7	7	897963	1	98.63	0.00	2

^a^ Number of CDS obtained from Prokka annotation

^b^ Calculated with CheckM

^c^ Classification according results of phylogenetic analyses (see Text)

^d^ Metagenome-assembled genomes. MAG1: *F*. *fructosus* isolate SRR10914162_bin.1_metawrap_v1.3.0_MAG genome assembly; MAG2: *F*. *fructosus* isolate SRR10810030_bin.2_metawrap_v1.3.0_MAG genome assembly; MAG3: *F*. *fructosus* isolate SRR12181049_bin.1_metawrap_v1.3.0_MAG genome assembly; MAG4: uncultured *Fructobacillus* sp. isolate SRR10914163_bin.14_metawrap_v1.3.0_MAG genome assembly.

DNA extraction from the *Fructobacillus* sp. CRL 2054 strain was done using cells from a pure culture single colony; then, cells were washed and inoculated in FYP broth [6] with 20 g/L of fructose and 10 g/L of glucose at 30°C without shaking. Before the cells reached the stationary phase, they were centrifuged at 4000 x *g* for 10 min, and the cell pellet was resuspended in 500 μL of a cryopreservative liquid provided by the sequencing company. The resuspended cells were transferred into tubes with solid phase reversible immobilization (SPRI) beads, mixing by inversion 10 times. Beads were washed with extraction buffer containing lysozyme and RNase A, and incubated at 37°C for 25 min. Proteinase K and RNaseA were added and incubated at 65°C for 5 min. Genomic DNA was purified using an equal volume of beads and resuspended in EB buffer (10 mM Tris-Cl, pH 8.5). DNA was quantified in triplicate with the Quantit dsDNA HS assay (Thermo Fisher Scientific, Indianapolis, USA) in an Eppendorf AF2200 plate reader (Eppendorf, Mississauga, Canada).

### Genome sequencing and *de novo* assembly

The genomic DNA of the strain CRL 2054 was sequenced by using a whole genome shotgun (WGS) strategy by MicrobesNG (https://microbesng.com). Genomic DNA libraries were prepared using Nextera XT Library Prep Kit (Illumina, San Diego, USA), following the manufacturer’s protocol with few modifications: two nanograms of DNA were used as input, and PCR elongation time was increased to 1 min. DNA quantification and library preparation were carried out on a Hamilton Microlab STAR automated liquid handling system (Hamilton, Reno, USA). Pooled libraries were quantified using the Kapa Biosystems Library Quantification Kit for Illumina on a Roche light cycler 96 qPCR machine (Roche, Indianapolis, USA). Libraries were sequenced on the Illumina HiSeq using a 250 bp paired end protocol. In total, 404,773 paired-end sequenced reads were obtained, with 99x-fold coverage. Adapters were trimmed using Trimmomatic 0.30 [43]. The quality was assessed using in-house scripts combined with SAMTools, BEDTools and the BWA-MEM software. *De novo* assembly of reads was performed using SPAdes version 3.7 [44].

### Characterization and functional annotation of genomes

The assembly metrics and GC content (%) of the studied genomes were determined with QUAST [45]. Completeness and contamination percentages in all genomes were calculated with CheckM [46] using a set of marker genes for organisms belonging to the order *Lactobacillales*. A search for specific genes related with horizontal transfer elements, antimicrobial functions, and antibiotic resistance was also performed in these genomes (https://crisprcas.i2bc.paris-saclay.fr/CrisprCasFinder/Index; https://phaster.ca; https://isfinder.biotoul.fr; http://bagel4.molgenrug.nl; https://cge.cbs.dtu.dk/services/ResFinder-4.1). CRISPRCasFinder [47] and Phaster tool [48] were used to find and characterize putative CRISPR-Cas systems and prophage regions, respectively. ISfinder database [49] was used to identify genes related to insertion sequence (IS) elements. Moreover, the identification of bacteriocin-coding sequences was performed with BAGEL4 [50], and antibiotic resistance genes were found by comparison against ARG-ANNOT [51] and ResFinder [52] databases.

To keep uniformity in the analysis, the prediction of coding sequences (CDS) and functional annotation of genes in all genomes was done using Prokka [53]. Genomes were also annotated in the RAST server and its SEEDViewer tool was used to confirm the presence or absence of some genes of interest and their genomic context. To search for metabolic differences in the studied *Fructobacillus* organisms, the genes of the studied genomes were grouped into metabolic categories by comparison with the KEGG (Kyoto Encyclopedia of Genes and Genomes) and COG (Cluster of Orthologous Genes) databases, using the tools BlastKOALA [54], and eggNOG mapper [55], respectively. Furthermore, the dbCAN2 tool [56] was also used for the screening of genes related to sucrose metabolism and biosynthesis of exopolysaccharides. The obtained data and additional information from KEGG database were used to make a comparative analysis of the presence or absence of genes involved in the central metabolism of these bacteria.

### Phylogenetic and pangenome analyses

Three phylogenetic analyses were performed on *Fructobacillus* using different approaches. Initially, a phylogenetic tree was obtained based on the 16S rRNA sequences of *Fructobacillus* and related organisms. Metagenome-assembled genomes (*Fructobacillus* genomes MAG1 to MAG4), were excluded from this analysis, since the sequence of the 16rRNA gene was not found in these genomes. Sequences were aligned with ClustalW; poorly aligned regions were manually trimmed. The tree was obtained applying the Maximum-likelihood method with IQTREE [57]. The TIM3+F+R3 substitution model, previously determined as the best-fit substitution model for this dataset was used for tree inference. The root was fixed using the sequence of *Lactobacillus delbrueckii* subsp. *delbrueckii* BCRC 12195 as an outgroup member. Additionally, the identity percentages among the available 16S-rRNA sequences of *Fructobacillus* organisms were calculated performing a global alignment in CLUSTALOmega [58].

A second tree was designed using single-copy core genes present in all *Fructobacillus* organisms and *Leuconostoc mesenteroides* ATCC 8293^T^ (used as outgroup). Genes were aligned using MAFFT with default parameters and then concatenated. Poorly aligned regions were removed with Gblocks [59]. The best evolutionary model was determined for each codon position (1^st^, 2^nd^, or 3^rd^), and the tree was inferred with IQTREE [57] applying the Maximum-likelihood method. In both trees, the robustness of the branches was measured by ultrafast bootstrapping (UFB) of 10,000 replicates.

Finally, a third phylogenetic approach was performed to find the rate of recombinant sites respect to mutations in the *Fructobacillus* core genome. To this end, an initial Maximum-likelihood tree using the core genes of the studied genomes was obtained with IQTREE. Then, an analysis of recombinant sites in the core-genome alignment was done by ClonalFrameML software [60]. The *R/θ* parameter, known as the ratio of recombination to mutation, was calculated with the EM algorithm by performing 100 simulations.

To estimate the number of core and accessory genes in the studied genomes, groups of orthologous genes were identified through GET_HOMOLOGUES software [61]. The OrthoMCL algorithm was chosen to cluster genes in orthologous groups [62]. The default identity threshold was modified by up to 40% to fit a genus analysis.

### Statistical analyses

Wilcoxon’s non-parametric test (Mann–Whitney U) was applied to compare the number of genes involved in the COG categories and KEGG modules between the two *Fructobacillus* groups. Analyses were performed using the InfoStat Statistical Software (Universidad Nacional de Córdoba, Córdoba, Argentina). Furthermore, clustered heatmaps were obtained in all cases by using the Pheatmap R package.

## Results

### General characteristics of the studied *Fructobacillus* genomes

Twenty-four genomes of *Fructobacillus* strains were studied to identify genomic differences within this genus. NCBI accession numbers, along with genome features and assembly statistics are summarized in Table 1.

The genome of *Fructobacillus* sp. CRL 2054 was successfully sequenced with 99.07x coverage. The resulting draft sequence after the *de novo* assembly contained 1,326,779 bp divided into 28 contigs higher than 200 bp. The N50 parameter, related to the quality of assembly, was higher than 100 kb.

Only one of the studied genomes presented complete status (*Fructobacillus* sp. KI3_B9 –accession number CP097122.1), whereas the rest of the genomic sequences were fragmented into contigs or scaffolds (Table 1), 20 of them presenting less than 50 contigs. No plasmidic DNA was found in any of the studied strains. Despite the draft status of the majority of the studied genomes, all the sequences showed more than 97% completeness and low contamination (below 2%). These data indicate that all genomes present a near-complete status according to Parks et al. [46], and are suitable for the comparative genomic analysis.

In general, a small genome size was observed in the genomes of all studied *Fructobacillus* (1.15–1.75 Mbp). However, genomes of 14 strains (belonging to the species *durionis*, *fructosus*, *papyriferae*, *papyrifericola*, *broussonetiae*, *parabroussonetiae* and *apis*, and two sp. organisms) were considerably smaller (1.15–1.38 Mbp) than the rest of the genus, whereas genomes of *F*. *ficulneus*, *F*. *tropaeoli* and *F*. *cardui* strains were larger (1.54–1.75 Mbp). A wide range in G+C content (43.6–48.5%) was found throughout this genus. In particular, strains belonging to the recently descripted species *papyriferae*, *papyrifericola*, *broussonetiae*, *parabroussonetiae* and *apis*, along with *Fructobacillus* sp. M158, showed markedly higher GC content (46.1–48.5%) than the rest of the studied strains (43.7–45.0%).

### Phylogenetic analyses in *Fructobacillus*

**Fig 1:**
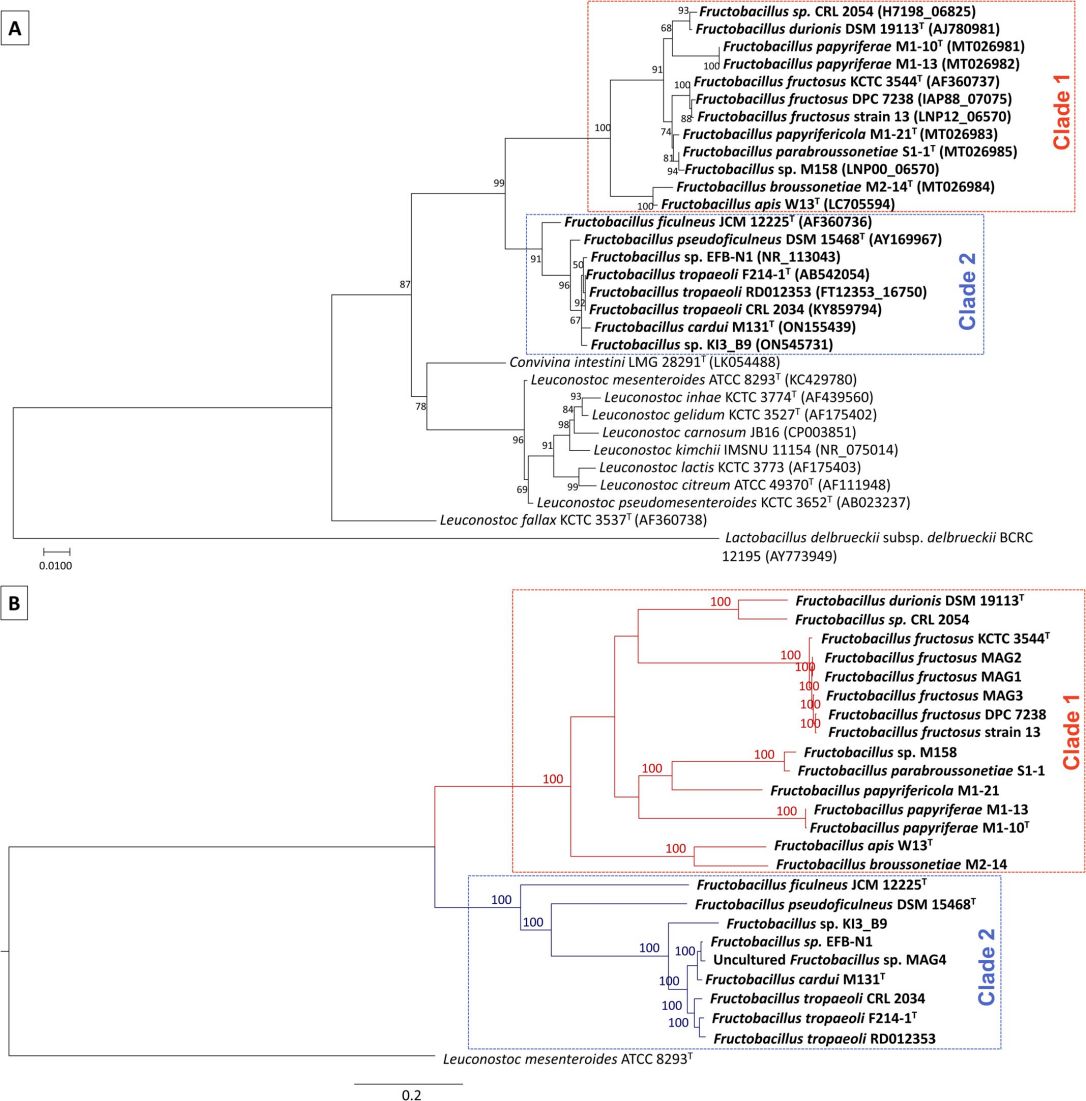
Phylogenetic trees showing the relationship between *Fructobacillus* organisms. **A:** Phylogenetic tree based on the 16S rRNA gene of *Fructobacillus* and related organisms. The sequence of *Lactobacillus delbrueckii* subsp. *delbrueckii* BCRC 12195 was used as outgroup. Accession numbers are indicated in parentheses besides taxa names. **B:** Maximum-likelihood phylogenetic tree based on the core genome of *Fructobacillus* and *L*. *mesenteroides* ATCC 8293^T^ (used as outgroup strain). Both trees were inferred by using the Maximum Likelihood method. Ultrafast bootstrap (UFB) percentages based on 10,000 replicates are given at branching points. Identified *Fructobacillus* clades 1 and 2 are highlighted in both trees.

**Fig 2:**
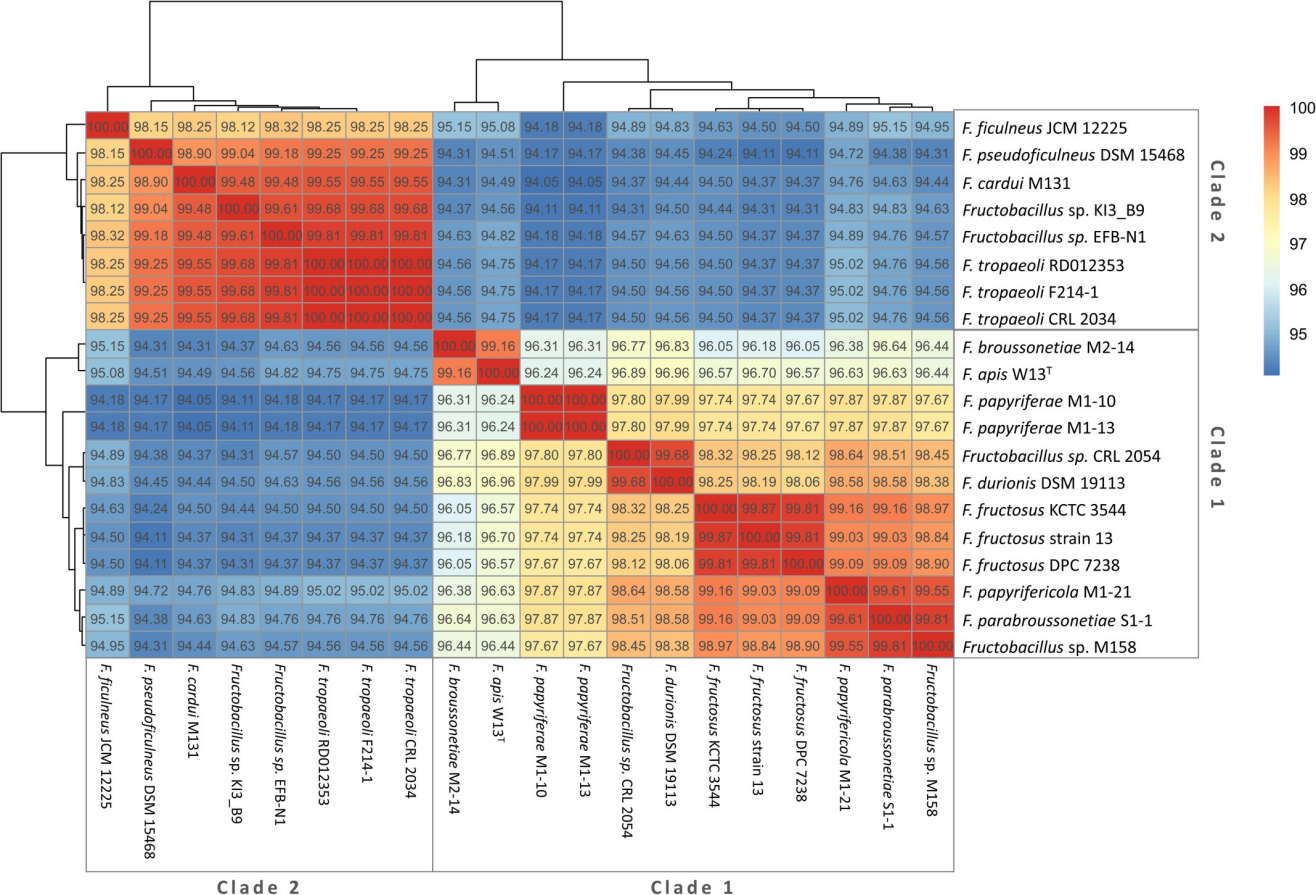
Clustered heatmap showing 16S- rRNA identity between pairs of *Fructobacillus* strains. Organisms with similar identity values are located in the same cluster.

A phylogenetic tree was made using 16S rRNA sequences of *Fructobacillus* and related organisms (Fig 1A). Two clades could be distinguished among the *Fructobacillus* sequences. The first clade was composed of *Fructobacillus* sp. CRL 2054, *F*. *durionis* DSM 19113^T^, *F*. *fructosus* strains, *F*. *papyriferae* strains, *F*. *papyrifericola* M1-21^T^, *F*. *broussonetiae* M2-14^T^, *F*. *parabroussonetiae* S1-1^T^, *F*. *apis* W13^T^ and *Fructobacillus* sp. M158, while the second clade consisted of *F*. *tropaeoli* strains, *F*. *pseudoficulneus* DSM 15468^T^, *F*. *ficulneus* JCM 12225^T^, *F*. *cardui* M131^T^, *Fructobacillus* sp. KI3_B9 and *Fructobacillus* sp. EFB-N1. According to this tree, the 16s rRNA sequence of the clade 1 showed a markedly higher divergence against its common ancestor when compared to the clade 2. In addition, the identity values of the 16S rRNA gene between pairs of strains were used to design a clustered heatmap (Fig 2). Organisms of the same phylogenetic clade were clustered together. The sequences presented more than 96% identity between organisms of the same clade and 94 to 95% identity between organisms of opposite clades, indicating that this gene is highly different between both groups. In the same way, another phylogenetic tree was inferred by the Maximum Likelihood method using 656 core genes present in *Fructobacillus* strains and *L*. *mesenteroides* ATCC 8293^T^ (used as outgroup) (Fig 1B). The resulting alignment (554,163 bp-long after trimming poorly aligned regions) was used for the tree inference. The two groups of strains already described were also located in different clades in this tree. In addition, *F*. *fructosus* MAG1 to MAG3 strains were located along with other *F*. *fructosus* strains in clade 1, whereas *Fructobacillus* sp. MAG4 belonged to clade 2. As previously observed in the 16S tree, members of the first clade were also more distant from the common ancestor than organisms belonging to the second clade.

Additionally, the events of homologous recombination in the core genome of the studied genomes were determined through a Maximum-likelihood approach by using the ClonalFrameML software. This approach allowed to identify 2132 recombinant events among the studied *Fructobacillus* genomes. The value of the *R/θ* parameter (ratio of recombination events respect to mutations) was 0.0112 (± SD: 1,43E-03), indicating that mutation events occur at roughly 90 times more than recombination in the core genome of the studied *Fructobacillus*.

### Identification of prophages, CRISPR-Cas9 systems and bacteriocin- and antibiotic resistance- encoding genes

Genomic regions related to horizontal gene transfer (HGT), antimicrobial properties, and antibiotic resistance in the *Fructobacillus* genomes were identified (Table 2). Only prophages with PHASTER score higher than 70 (complete or questionable state) were analyzed. At least one prophage region was present in nineteen out of the twenty-four genomes, these regions were present in organisms of both phylogenetic clades described before. Twelve genomes contained one prophage, whilst seven genomes contained two or more prophage regions. Of all 28 prophages identified, nineteen regions were intact (18–55 kb in size), and nine contained partial regions (15–33 kb), which were usually located at the start or end of a contig (S1 Table). All prophages presented similar GC content (34.0–43.4%). Genes coding for a terminase, protease, coat protein, portal protein, tail shaft, and other phage-related proteins represented more than 50% of total genes, while the rest were not associated with any known function. A gene coding for a plate protein was only found in one of the prophages of *F*. *tropaeoli* F214-1^T^. Furthermore, an attachment site, which is necessary for phage insertion into the bacterial chromosome, was observed in fourteen of the studied prophages. Moreover, different tRNA genes were found in six prophages. A BLASTn search showed certain similarities between nineteen of the twenty-eight *Fructobacillus* prophages and sequences of other phages, previously found in the metagenome of a honeybee [63]; however, the alignment coverage percentage was low (< 60%) for most of the studied sequences, indicating that most of the *Fructobacillus* prophages have not been previously described. In most cases, the *Fructobacillus* prophage sequences (with the exception of one of the prophages of *F*. *tropaeoli* F214-1 and *F*. *cardui* M131) were similar to sequences of the *Siphoviridae* family viruses (S1 Table).

**Table 2:** Genomic regions related to horizontal transfer elements and antimicrobial properties found in *Fructobacillus* studied genomes.

Strains	Clade	Prophages*	CRISPR-Cas systems	Bacteriocins- CDS	IS elements
*Fructobacillus* sp. CRL 2054	1	2 intact (32 and 33 kb)	No	No	No
*F*. *durionis* DSM 19113^T^	1	No	No	Yes	No
*F*. *fructosus* KCTC 3544^T^	1	1 questionable (contig start) (16 kb)	Yes (Cas-type IIa, 8 spacers)	No	3 IS transposases
*F*. *fructosus* DPC 7238	1	1 intact (24 kb)	Yes (Cas-type IIa, 7 spacers)	No	3 IS transposases
*F*. *fructosus* strain 13	1	1 questionable (contig start) (18 kb)	No	No	2 IS transposases
*F*. *fructosus* MAG1	1	1 intact (37 kb)	No	No	3 IS transposases
*F*. *fructosus* MAG2	1	2 intact (25 and 33 kb)	No	No	1 IS transposase
*F*. *fructosus* MAG3	1	No	No	No	1 IS transposase
*F*. *papyriferae* M1-10^T^	1	1 intact (55 kb)	No	No	No
*F*. *papyriferae* M1-13	1	1 intact (55 kb)	No	No	No
*F*. *papyrifericola* M1-21^T^	1	1 intact (44 kb)	No	No	No
*F*. *broussonetiae* M2-14^T^	1	No	No	No	No
*F*. *parabroussonetiae* S1-1^T^	1	No	No	No	1 IS transposase
*Fructobacillus* sp. M158	1	1 intact (18 kb); 2 questionable (17 and 13 kb)	No	No	1 IS transposase
*F*. *apis* W13	1	1 intact (31 kb)	No	No	2 IS transposases
*F*. *ficulneus* JCM 12225^T^	2	1 intact (30 kb)	Yes (1 Cas-type IIa system with 5 spacers; 5 spacers without Cas (short contig); 4 spacers without Cas (contig start))	No	5 IS transposases
*F*. *pseudoficulneus* DSM 15468^T^	2	No	Yes (Cas-type IIa, 4 spacers (end of contig))	No	3 IS transposases
*Fructobacillus* sp. EFB-N1	2	1 intact (23 kb), 1 questionable (contig start) (17 kb)	Yes (Cas-type IIa, 3 spacers)	No	11 IS transposases
*F*. *tropaeoli* F214-1^T^	2	2 intact (21 and 24 kb)	No	No	3 IS transposases
*F*. *tropaeoli* CRL 2034	2	1 questionable (contig start) (33kb), 1 intact (36 kb)	No	No	1 IS transposase
*F*. *tropaeoli* RD012353	2	1 intact (40 kb)	Yes (Cas-type IIa, 11 spacers)	No	5 IS transposases
*F*. *cardui* M131^T^	2	1 intact (40 kb); 2 questionable (contig start) (30 and 18 kb)	No	No	2 IS transposases
*Fructobacillus* sp. KI3_B9	2	1 intact (36 kb)	No	No	1 IS transposase
*Fructobacillus sp*. MAG4	2	1 questionable (15 kb)	Yes (Cas-type IIa, 7 spacers)	No	4 IS transposases

*Prophages were classified according to scores assigned by PHASTER (Intact: 90–150, Questionable: 70–90).

CRISPR-Cas systems, widely distributed in bacteria to provide immunity against foreign DNA, were found in seven out of the twenty-four studied genomes (Table 2). Two of the *Fructobacillus* genomes harboring these systems belonged to clade 1 (*F*. *fructosus* strains), while the rest of the genomes were part of clade 2. According to the CRISPR-Cas systems classification, all identified regions belonged to type IIa Cas systems, presenting genes coding for Cas9, Cas1, Cas2, and Csn2 proteins. A variable number of spacer sequences (4 to 11) between short palindromic repeats were found downstream of these genes. Genomes of *F*. *fructosus* strains and *F*. *tropaeoli* RD012353 harbored the highest number of spacers (between 7 and 11). *F*. *ficulneus* JCM 12225 also presented two sets of CRISPR repeats without Cas genes that were truncated by a contig start.

Insertion sequence (IS) elements, mobile elements of short length (0.7–2.5 kb) that contain genes coding for transposases, responsible for the insertion of these DNA segments in the bacterial genome [64], have been also sought. Several IS transposases, mainly those belonging to IS3 and IS30 families were widespread in the *Fructobacillus* genomes. *Fructobacillus* sp. EFB-N1 stood out containing 11 different transposases, being this value clearly higher than those observed in the other genomes, which contained 0 to 5 transposases only.

The identification of genes related to antimicrobial activity in *Fructobacillus* organisms was also performed. Only *F*. *durionis* DSM 19113^T^ harbored two contiguous regions encoding two peptides of a bacteriocin. These genes showed similarity against peptide chain A (59.52% identity) and peptide chain B (51.67% identity) of the bacteriocin LS2, a member of the class IId bacteriocins produced by *Ligilactobacillus salivarius* BGH01. A gene coding for an ABC transporter (necessary for peptide export) was also found in this strain near to the bacteriocin- coding genes.

The search for antibiotic resistance genes against the ARG-ANNOT and ResFinder databases showed that *Fructobacillus* genes did not have significant similarities with antibiotic resistance genes present in the aforementioned databases. However, according to the genome annotation previously obtained with Prokka, seven different genes classified as multidrug resistance genes were identified in the pangenome, from which three *emrB* genes encoding a multidrug exporter and a gene related with the quaternary ammonium-compound resistance were present in all genomes. Furthermore, two genes involved in the resistance to byciclomycin and disinfectants of the family of quaternary ammonium compounds were only detected in the genomic sequence of *F*. *tropaeoli* F214-1^T^.

### Pangenome analysis of *Fructobacillus*

**Fig 3:**
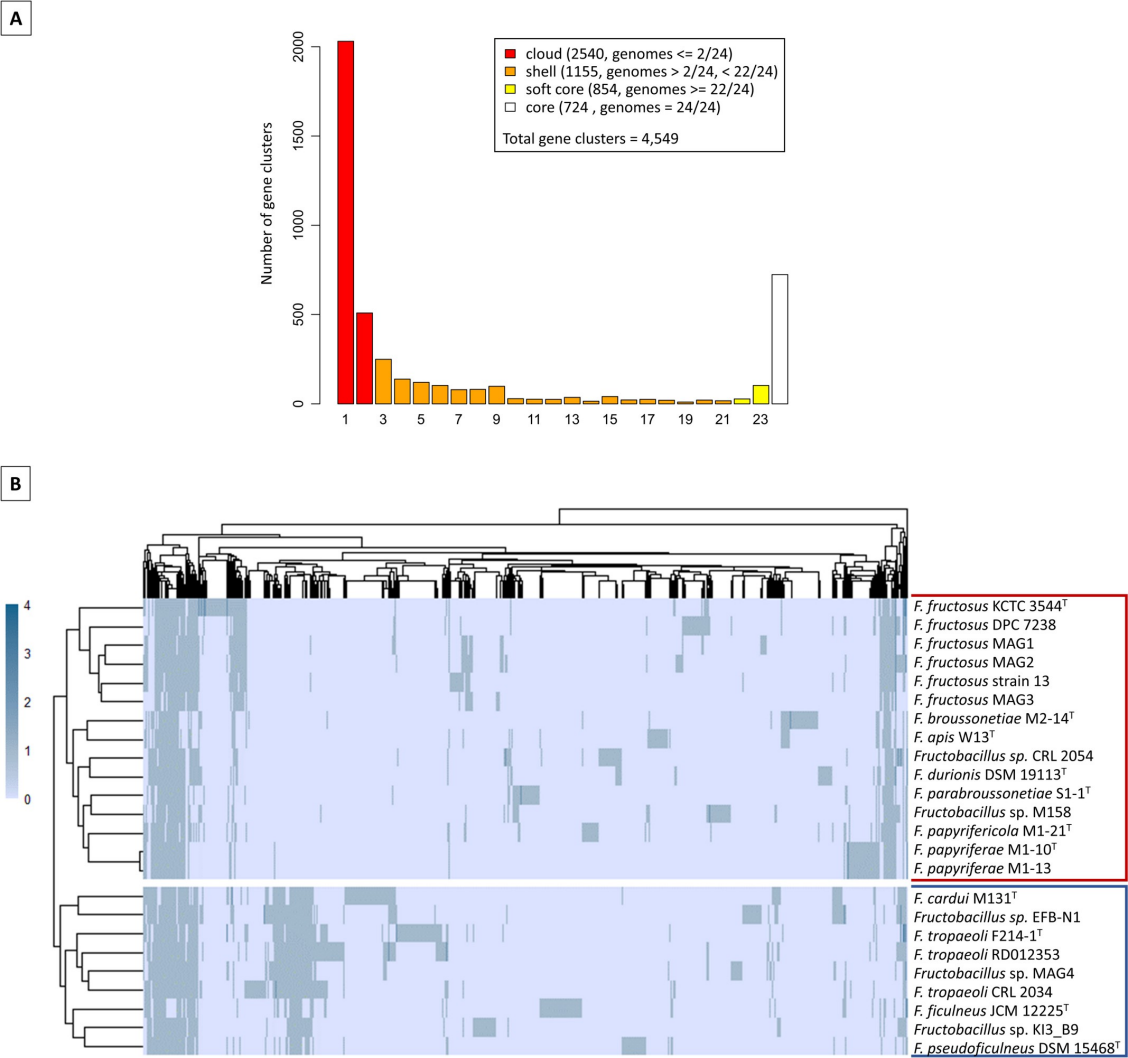
Pangenome analyses. **A:** Bar graph showing the number of gene clusters present in 1 to 24 *Fructobacillus* genomes. The color of the bars represents the cloud, shell, soft-core, or core genes. **B:** Clustered heatmap based on the presence (blue bars) or absence (light blue bars) of gene families in the dispensable genome of *Fructobacillus* strains. Organisms belonging to clades 1 or 2 of the phylogenetic analyses are enclosed with a red (clade 1) or blue (clade 2) rectangle.

For this study, genes from the twenty-four studied genomes were clustered into orthologous groups. This analysis resulted in 4,549 gene clusters, which make up the pangenome of this set of *Fructobacillus* genomes. Out of the total detected groups, 724 genes were present in all genomes (core genome), and 854 genes were found in 22 genomes or more (soft-core genome). Furthermore, 3,695 groups of genes were part of the dispensable genome, of which 1,155 were present in 3 to 21 genomes (shell genome), and 2,540 were each located in one or two strains (cloud or unique genome). Interestingly, the genes present in 3 to 9 genomes constituted an important part of the shell (868 out of 1,155 genes) (Fig 3A).

The results of the pangenome analysis were also used to cluster the studied organisms based on the presence or absence of genes in the dispensable genome. As shown in Fig 3B, organisms were also clustered in two opposite clades, being this division identical to that observed in the phylogenetic analyses. Moreover, a noticeable number of genes were present in members of clade 2 and absent in strains of clade 1. In the same way, a lower number of genes were present in all genomes of the first clade and in none of the second clade genomes. These findings evidence a clear difference in the genetic content among the studied *Fructobacillus* strains.

### Characterization of *Fructobacillus* genomes through classification in COG categories and KEGG metabolic modules

**Fig 4:**
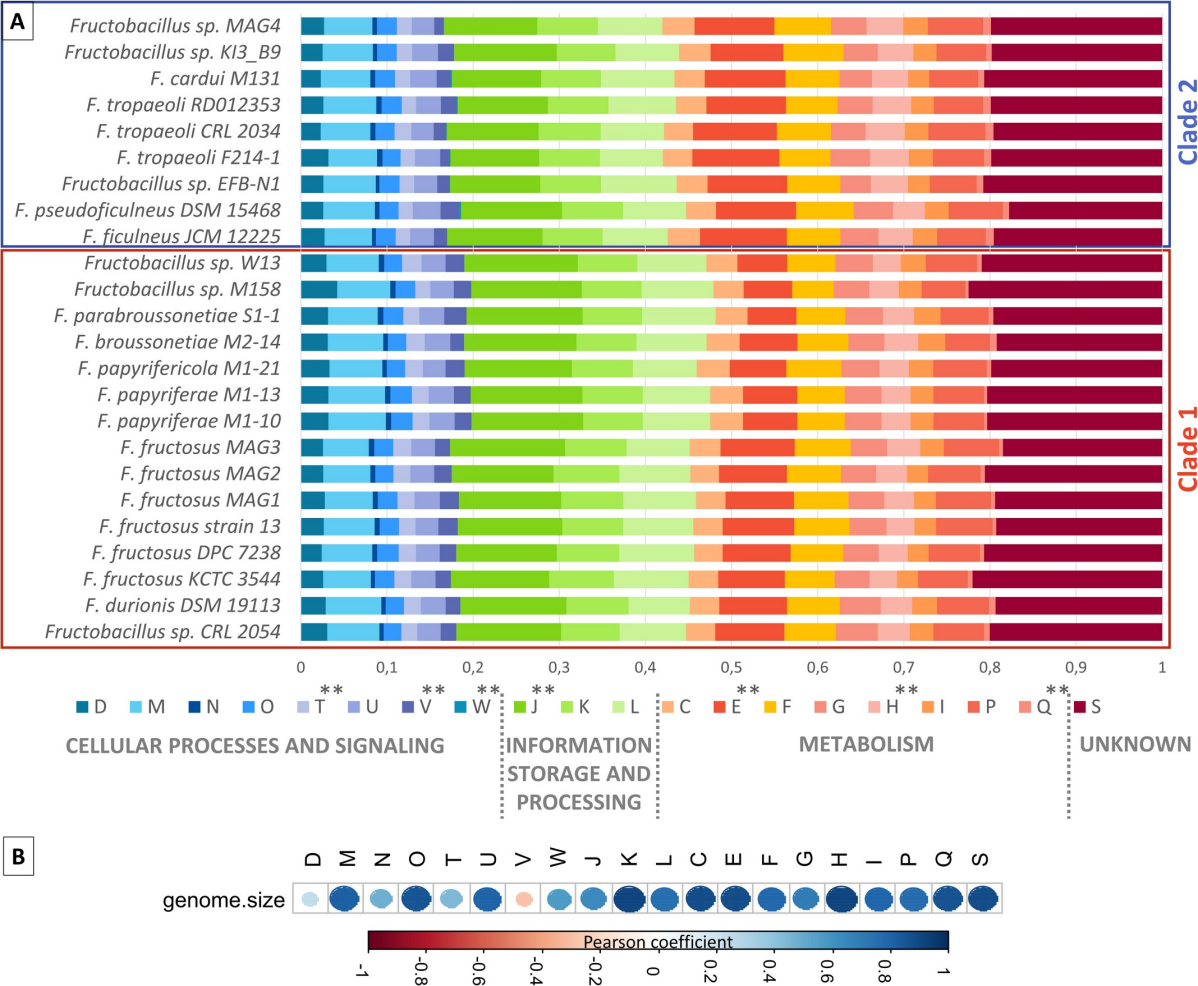
Functional annotation of genes in COG categories. **A:** Comparative analysis of classification of genes in COG categories among *Fructobacillus* genomes. Each part of stacked bars represents the ratio of genes in each COG category vs. the total number of COG-annotated genes for each *Fructobacillus* strain. Members of each clade of the phylogenetic analyses are highlighted in the figure. Categories presenting significant differences between both groups (clades 1 and 2) are marked with one asterisk (p < 0.05) or two asterisks (p < 0.01). **B:** Correlation matrix between the number of genes in each COG category and the genome size of *Fructobacillus* organisms. The color in each circle represents the value of the Pearson correlation coefficient (blue: positive correlation, red: negative correlation). A COG category with a Pearson coefficient near to 1 indicate a strong positive correlation between the number of genes in that category and the genome size in the genus. Each COG category is represented with a letter. D: Cell cycle control, cell division, chromosome partitioning; M: Cell wall/membrane/envelope biogenesis; O: Post-translational modification, protein turnover, and chaperones; T: Signal transduction mechanisms; U: Intracellular trafficking, secretion, and vesicular transport; V: Defense mechanisms; W: Extracellular structures; J: Translation, ribosomal structure and biogenesis; K: Transcription; L: Replication, recombination and repair; C: Energy production and conversion; E: Amino acid transport and metabolism; F: Nucleotide transport and metabolism; G: Carbohydrate transport and metabolism; H: Coenzyme transport and metabolism; I: Lipid transport and metabolism; P: Inorganic ion transport and metabolism; Q: Secondary metabolites biosynthesis, transport, and catabolism; S: Function unknown.

Genes from all genomes were grouped into COG categories related to cellular processes and signaling, information storage and processing, metabolic processes, and unknown function (Fig 4A). The ratio of genes assigned in each COG category against the total number of genes in all COGs was determined for each strain and shown in Fig 4. Ratio values between clades 1 and 2 were compared for each COG category by performing the Wilcoxon test. The ratios of genes in organisms of clade 1 were significantly lower than in clade 2 in three categories related to metabolism [E (Amino acid transport and metabolism); H (Coenzyme transport and metabolism) and Q (Secondary metabolites biosynthesis, transport, and catabolism)] and in W category (related with extracellular structures). On the contrary, clade 1 presented higher gene ratios than clade 2 in T (Signal transduction mechanisms), V (Defense mechanisms), and J (Translation, ribosomal structure, and biogenesis) categories.

As described above, differences in size were observed in the analyzed genomes. In this way, a possible association between the number of genes in each COG category and genome sizes was assessed by calculating the Pearson correlation coefficient (Fig 4B). The highest positive correlation values (Pearson coefficient > 0.88) were found in the metabolism-associated categories E and H (involved in the transport and metabolism of amino acids and coenzymes, respectively), and in categories C (Energy production and conversion), and S (function unknown). These results could indicate a narrow relationship between the genome size of the studied *Fructobacillus* strains and their number of genes associated with the metabolism of nitrogen compounds and other cellular processes.

**Fig 5:**
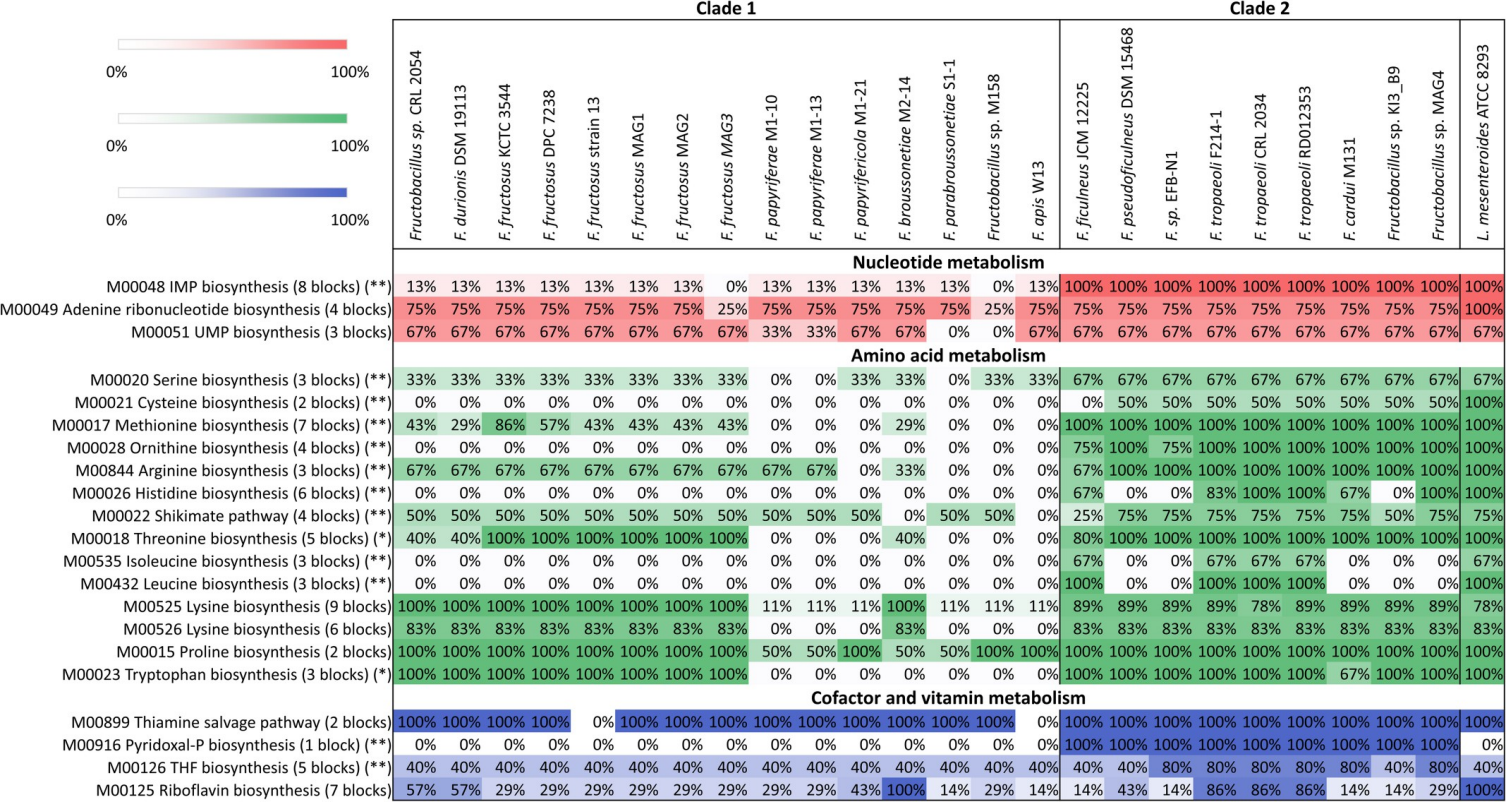
Pseudo-heatmap representing the level of completeness of KEGG metabolic modules in the studied *Fructobacillus* strains and *L*. *mesenteroides* ATCC 8293^T^. Numbers in parentheses indicate the number of total blocks for each pathway, and percentages show the ratio between found blocks in each genome and total blocks for each metabolic module. One asterisk (*p* < 0.05) or two asterisks (*p* < 0.01) indicate modules with significant differences in the number of present blocks between both groups of strains (clades 1 and 2).

The genes of each genome were also classified into metabolic modules using the KEGG mapper–Reconstruct pathway tool from the KEGG database. Complete and almost complete modules were selected for a comparative analysis among the *Fructobacillus* genomes. Percentages of completeness of each metabolic module in each strain are shown in Fig 5. Data of *L*. *mesenteroides* ATCC 8293^T^ was also included in the figure to compare the results with a related organism. The number of present blocks (genes or groups of genes forming part of one step in the pathway) were statistically compared between clades for each metabolic module. Large differences were distinguished in metabolic pathways related to amino acid biosynthesis. Genomes of the clade 1 presented a significantly lower number of genes (*p* < 0.05) associated with the biosynthesis of eleven amino acids (serine, cysteine, methionine, ornithine, arginine, histidine, shikimate, threonine, isoleucine, leucine, and tryptophan) when compared with organisms of the clade 2. Both groups also differed in the number of genes involved in the metabolism of cofactors and vitamins; organisms located in the first clade had significantly fewer genes related to the biosynthesis of tetrahydrofolate and pyridoxal-P. Furthermore, a remarkable difference between both groups in the biosynthesis of the inosine monophosphate nucleotide was also observed, where none or only one of the 8 blocks required for this pathway was found in organisms of the clade 1. Furthermore, the level of completeness of each metabolic module was usually similar between genomes of the second clade and *L*. *mesenteroides* ATCC 8293^T^. These results confirm a lack in the synthesis of several amino acids and some vitamins and nucleotides in organisms belonging to clade 1 with respect to members of the second clade and the type strain of *L*. *mesenteroides*.

### Analysis of genes involved in the central metabolism in *Fructobacillus*

**Fig 6:**
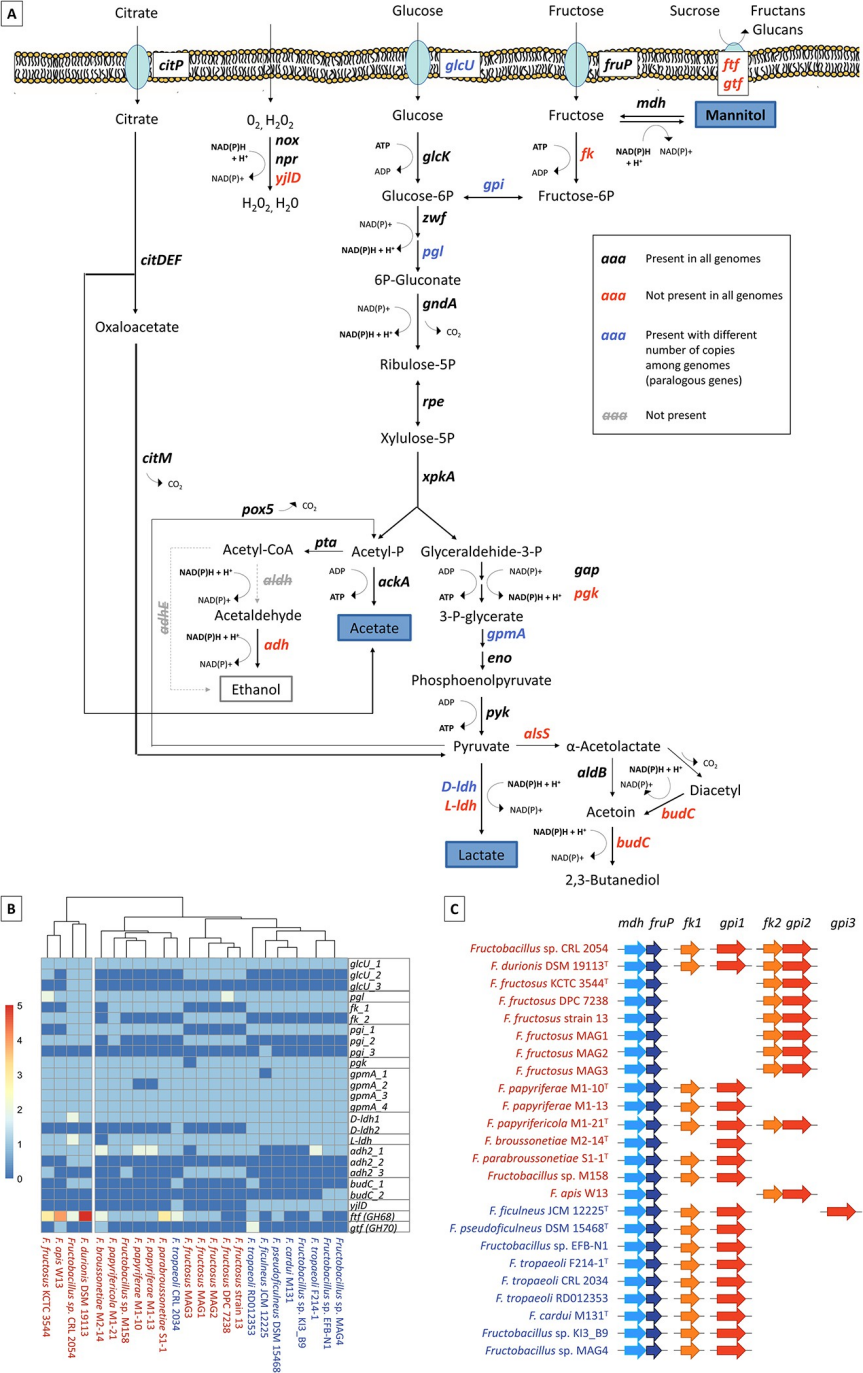
Differences in genes involved in the central metabolism and the use of fructose among *Fructobacillus* genomes. **A:** Predicted metabolic pathways related to carbohydrate metabolism and the use of electron acceptors in *Fructobacillus* [adapted from Ruiz Rodriguez et al. (2020)]. Genes that are not present in all the studied genomes (differences in presence/absence) are shown in red, while genes that present differences in the number of copies (paralogous genes) are colored in blue. **B:** Clustered heatmap of genes of the central metabolism showing differences in presence/absence among the studied *Fructobacillus* strains. Strains belonging to clade 1 or 2 are indicated in red or blue, respectively. **C:** Schematic representation of the distribution of genes related to the use of fructose among the studied strains. Genes involved in the use of fructose as an electron acceptor are represented as light blue and blue arrows (*mdh* and *fruP*, respectively); whereas genes related to the use of fructose as an energy substrate are shown with orange and red arrows (*fk* and *gpi*, respectively). *glcU*: Putative glucose uptake permease; *fruP*: Putative fructose permease; *glcK*: Glucokinase (EC 2.7.1.2); *zwf*: Glucose-6-phosphate 1-dehydrogenase (EC 1.1.1.49); *pgl*: 6-phosphogluconolactonase (EC 3.1.1.31); *gndA*: 6-phosphogluconate dehydrogenase, (EC 1.1.1.44); *rpe*: Ribulose-phosphate 3-epimerase (EC 5.1.3.1); *xpkA*: Xylulose-5-phosphate phosphoketolase (EC 4.1.2.9); *gpi*: Glucose-6-phosphate isomerase (EC 5.3.1.9); *fk*: Fructokinase (EC 2.7.1.4); *mdh*: Mannitol 2-dehydrogenase (EC 1.1.1.14); *gap*: Glyceraldehyde-3-phosphate dehydrogenase (EC 1.2.1.12); *pgk*: Phosphoglycerate kinase (EC 2.7.2.3); *gpmA*: Phosphoglycerate mutase (EC 5.4.2.1); *eno*: Enolase (EC 4.2.1.11); *pyk*: Pyruvate kinase (EC 2.7.1.40); D-*ldh*: D-lactate dehydrogenase (EC 1.1.1.28); L-*ldh*: L-lactate dehydrogenase (EC 1.1.1.27); *pta*: Phosphate acetyltransferase (EC 2.3.1.8); *adh*: Alcohol dehydrogenase (EC 1.1.1.1); *pox5*: Pyruvate oxidase (EC 1.2.3.3); *pdc*: Alpha-keto-acid decarboxylase (EC 4.1.1.-); *ackA*: Acetate kinase (EC 2.7.2.1); *alsS*: Acetolactate synthase, catabolic (EC 2.2.1.6); *aldB*: Alpha-acetolactate decarboxylase (EC 4.1.1.5); *budC*: 2,3-butanediol dehydrogenase, S-alcohol forming, (R)-acetoin-specific (EC 1.1.1.4)/Acetoin (diacetyl) reductase (EC 1.1.1.5); *npr*: NADH peroxidase Npx (EC 1.11.1.1); *nox*: NADH oxidase; *yjlD*: NADH dehydrogenase (EC 1.6.99.3); *ftf*: fructosyltransferases (Glycoside Hydrolase Family 68); *gtf*: glucosyltransferases (Glycoside Hydrolase Family 70).

The information retrieved from the KEGG database and the results of pangenome studies were used to reconstruct the central metabolic pathway in *Fructobacillus* organisms, considering the metabolism of carbohydrates and the use of electron acceptors for the maintenance of redox balance. Additionally, a comparative description was made by analyzing differences in genes involved in the central metabolism among the genomes under study (Fig 6A). Genes for the synthesis of lactate, acetate and carbon dioxide were identified. The 6-phosphogluconate/phosphoketolase pathway, present in heterofermentative LAB, was almost complete in these organisms, with the exception of the bifunctional *adhE* gene (involved in the reduction of acetyl-CoA to ethanol through acetaldehyde dehydrogenase and alcohol dehydrogenase activities). The lack of this gene is a feature of the fructophilic behavior of these organisms. Nevertheless, three different families of *adh* genes with alcohol dehydrogenase activity (EC 1.1.1.1), necessary for ethanol synthesis through acetaldehyde reduction, were distributed in 20 *Fructobacillus* genomes. Genes encoding for acetaldehyde dehydrogenases were not detected.

Differences in the presence/absence or in the copy number of genes among *Fructobacillus* genomes were observed in 14 out of total 38 genes involved in central metabolism (Fig 6A). Nine of these genes (*fk*, L-*ldh*, *alsS*, *pgk*, *budC*, *adh*, *yjlD*, fructosyltransferases and glucosyltransferases) were only present in some of the studied genomes, whereas five genes (*glcU*, *pgi*, *pgl*, *gpmA* and D-*ldh*) differed in the number of copies (paralogous genes) among *Fructobacillus* organisms. Furthermore, from the initial fourteen genes presenting differences among genomes, eight of these were strictly related to the use of fructose and electron acceptors. Within this group, six genes (*yjlD*, *adh*, D-*ldh*, L-*ldh*, *alsS*, and *budC*) were associated with the use of electron acceptors and NAD^+^ regeneration, while two genes (*fk* and *gpi*) were involved in the use of fructose as a growth substrate. In addition, two genes (the paralogous gene D-*ldh2* and the NADH dehydrogenase gene *yjlD*) were only present in the genomes of the second clade. Noteworthy, the presence or absence of most genes of the central metabolism was not clade-specific. As observed in Fig 6B, organisms of each phylogenetic clade were widely distributed in different clusters according to the presence/absence of central metabolism- genes, indicating that differences observed in genes of the central metabolism are not related with the phylogeny among organisms. For instance, the *budC* gene, which is involved in the regeneration of NAD(P)^+^ through reduction of diacetyl and acetoin, was only present in two out of the three members of *F*. *tropaeoli* species.

Seventeen *Fructobacillus* genomes harbored genes with specific domains of fructosyltransferases (*ftf–*GH68 family), which includes levansucrases and other enzymes involved in the synthesis of different types of fructans that use sucrose as their preferential donor substrate. In the same way, only three genomes (*F*. *tropaeoli* RD012353, *F*. *broussonetiae* M2-14^T^, and *F*. *apis* W13) presented genes with specific domains of glucosyltransferases (*gtf–*GH70 family), involved in the synthesis of dextran and other glucans from sucrose (Fig 6B).

Pyruvate can be used for NAD(P)H reoxidation through reduction to lactate by lactate dehydrogenases (LDHs), or by synthesis of aroma compounds (diacetyl and acetoin). Two D- lactate dehydrogenase genes, named D-*ldh1* and D-*ldh2*, were identified in *Fructobacillus* organisms of clade 2. Although genomes of the clade 1 only contained the D-*ldh1* gene, the strain *Fructobacillus* sp. CRL 2054 stood out by harboring two identical copies (100% identity) of D-*ldh1* with its ribosomal binding site (RBS). Moreover, most strains contained one copy of a putative L-*ldh* gene in their genomes; however, *F*. *durionis* DSM 19113^T^ presented two contiguous L-*ldh* genes while *F*. *broussonetiae* M2-14^T^ did not harbor any copy. Regarding pyruvate reduction through the synthesis of aroma compounds, the *alsS* gene (related to the conversion of pyruvate into alpha-acetolactate) and the *budC* gene (responsible for NAD(P)H reoxidation in this pathway) were found in 20 strains and 6 strains, respectively; the presence of *budC* being strain-specific (Fig 6B). In addition, the required genes for the synthesis of pyruvate for later use as electron acceptor through the assimilation of citrate were present in the core genome of *Fructobacillus* (Fig 6A).

Oxygen can also be used as an electron acceptor, being reduced to H_2_O with H_2_O_2_ as an intermediate. Three NADH oxidase genes were identified in *Fructobacillus*, and two of them were included in the core genome. However, the *yjlD* gene coding for a NADH dehydrogenase-like protein was only found in genomes belonging to the second clade.

Fructose can be used by these organisms as an electron acceptor or as a growth substrate. The reduction of fructose to mannitol is performed by the MDH, whereas the fructokinase (FK) and glucose-6-phosphate isomerase (GPI) enzymes are used for the assimilation of fructose in heterofermentative LAB. A schematic representation of the distribution of genes related to the use of fructose is shown in Fig 6C. The MDH gene (*mdh*) and a putative fructose non-phosphorylating permease gene (*fruP*) were located contiguously in all *Fructobacillus* genomes (Fig 6C). On the contrary, two *fk* genes (*fk1* and *fk2*) and three *gpi* genes (*gpi1*, *gpi2* and *gpi3*) were differentially distributed in the studied strains. The *fk1* and *gpi1* genes were found in different parts of the genomes of all fructobacilli, except for the *F*. *fructosus* and *F*. *apis* strains (where *fk2* and *gpi2* were present) and in *F*. *broussonetiae* M2-14^T^, where no *fk* gene was identified according to Prokka and RAST annotations. On the other hand, *fk2* and *gpi2* were present in some organisms (*F*. *fructosus* strains, *F*. *apis* W13, *F*. *durionis* DSM 19113 and *Fructobacillus* sp. CRL 2054), being contiguously located in these genomes. Another gene called *gpi3* was only detected in *F*. *ficulneus* JCM 12225^T^ (Fig 6C).

## Discussion

The genus *Fructobacillus* has been described as a group of FLAB microorganisms formerly belonging to the genus *Leuconostoc* that suffered a reduction in their genomes as a consequence of their adaptation to fructose-rich niches. *Fructobacillus* genomes present fewer genes involved in the metabolism of carbohydrates than other LAB, especially due to the lack of transporters of the phosphotransferase systems (PTS) [38]. Despite these previous findings, more information on the genomic properties of *Fructobacillus* is needed to improve the knowledge on multilevel system regulation and provide a better understanding of their behavior [22]. Our study is the first comparative genomic analysis of the *Fructobacillus* genus that includes genomes of the recently described species after February 2022 (*papyriferae*, *papyrifericola*, *broussonetiae*, *parabroussonetiae*, *cardui*, and *apis* species). Phylogenetic relationships were reconstructed using the 16S rRNA as well as the concatenated DNA sequences of 656 core *Fructobacillus* genes. These phylogenetic trees allowed the clear identification of two well-supported clades, as previously reported [1, 11, 38]. Several differences related to sequence similarity, general genomic properties, and gene content between the two clades were observed and further characterized. Regarding sequence similarity, the 16S rRNA identity showed lower values in genomes of opposite clades than those from the same group. Differences in the identity of the 16S ribosomal gene were around 6% for genomes of different groups, this value being the maximum allowed for organisms of the same genus [65]. A similar division of genomes in two clades was observed between the phylogenetic analyses and the clustering based on presence/absence of genes. No plasmids were found in the studied genomes, although lysogeny was widespread and several classes of mobile elements were present in the twenty-four *Fructobacillus* strains. However, the calculated *R/θ* values (ratio of recombination events respect to mutations) showed that the rate of homologous recombination events in the *Fructobacillus* core genome was around 90-fold lower than mutation events, indicating that HGT did not meaningfully affect the genetic content of each clade.

Regarding general genomic properties, most genomes of the clade 1 had smaller sizes than most genomes of the second clade, indicating genome reduction and the consequent decrease in the total number of genes in organisms of the first group. This latter feature was studied in depth by performing a comparative gene functional analysis based on the COG and KEGG databases. Results suggest a reductive evolution in organisms of clade 1, with fewer genes mainly involved in the metabolism of nitrogen compounds; more specifically, in the synthesis of several amino acids, coenzymes and some nucleotides. Several theories have attempted to explain reductive evolution, especially in insect endosymbiotic bacteria and in free-living marine cyanobacterial populations [66]. Of these hypotheses, a higher mutation rate appears to be a key factor for genome reduction in various prokaryotic lineages [67]. According to this theory, organisms with a high mutation rate (called “mutators”) can rapidly acquire beneficial mutations as a mechanism of adaptation to environmental changes. Such increases can also lead to further gene loss of dispensable functions. In insect-associated bacteria (such as *A*. *kunkeei* and *F*. *fructosus*) vertical transmission to insect offspring causes bottlenecks in their population structure, which leads to the fixation of deleterious mutations [67, 68]. In this work, the calculated *R/θ* parameter indicated that in *Fructobacillus* mutation events occur more often (90X) than recombination. Furthermore, phylogenetic analyses based on different targets (16S rRNA and core genes) showed a greater genetic distance of clade 1 from the common ancestor of *Fructobacillus* than clade 2, indicating more mutations accumulated in the first group that could be related to the reduction of their genomes. A noticeable mutational bias towards deletions was observed in bacteria in comparison to eukaryotes [69, 70]. Therefore, mutations in group 1 would have triggered the loss of several genes for the synthesis of nitrogenous compounds since these are usually available in fructophilic niches (such as fruits, flowers, and the gastrointestinal tract of honeybees). Amino acids and vitamins are usually present in fruits and other plant structures [71]. In the case of the intestinal microbiota of bees, these compounds can be provided by other bacterial members of the same habitat that possess the machinery for the synthesis of all amino acids, such as *Gilliamella* spp. and *Snodgrassella* spp. [72]. However, a more exhaustive study with a higher number of *Fructobacillus* genomes is necessary to confirm a reductive evolution affecting the nitrogen compounds biosynthesis in part of this genus.

Other authors have already described a decrease in the number of genes of the metabolism of nitrogenous compounds in other LAB. An earlier genomic study on *Apilactobacillus kunkeei* and *Fructilactobacillus sanfranciscensis* revealed significant gene loss in these species compared to other taxa. From the total lost genes with assigned functions, 22% affected amino acid metabolism, in particular, amino acid biosynthesis [68]. According to these authors, the results are consistent with a shift to a nutritionally-rich growth habitat, such as the gastrointestinal tract of honeybees. In addition, functional differences in gene clusters for proline, tryptophan, leucine, and arginine biosynthesis were observed among *A*. *kunkeei* strains, while genes for purine and pyrimidine biosynthesis were lost in one of the studied strains. The identified biosynthetic gene clusters were located in the same genomic regions in all strains, indicating independent losses [68]. Recently, Maeno et al. [73] suggested that amino acid and carbohydrate metabolism/requirement is highly variable among species of the family *Lactobacillaceae* (including the genus *Fructobacillus*). They observed a relatively large gradient (61.6) between the number of genes assigned to the COG class E (amino acid transport and metabolism) and the genome size of 174 *Lactobacillaceae* strains. On the other hand, despite the genomic differences observed in the nitrogen compound- biosynthesis in *Fructobacillus*, no clear correlation was found between the identified phylogenetic groups in this genus and the reported habitats for each species, recently reviewed by Filannino et al. [22]. Further studies are needed to elucidate if differences in nitrogen metabolism within *Fructobacillus* genus would impact in the ability to ferment matrices with low content of nitrogen compounds.

Other differences in gene content were found among *Fructobacillus* genomes, particularly in the central metabolism. However, most genes showed a clade-independent scattered pattern among strains. These genes were mainly focused on steps of NAD(P)^+^ recycling and catabolism of fructose, which indicates the importance of these processes in fructophilic LAB. Duplication events were already observed in *ldh* genes, as previously described by Bleckwedel et al. [74]. These authors observed high sequence similarity between paralogous genes *ldh1* and *ldh2* (74% identity), being *ldh1* the main responsible of the LDH activity in *F*. *tropaeoli* CRL 2034. An identical duplication (100% identity) of the *ldh1* gene and its RBS was also detected in *Fructobacillus* sp. CRL 2054, indicating a recent duplication event in this organism. This observation shows again the importance of duplication of *ldh* genes in *Fructobacillus*, although the reason of this phenomenon remains unknown. Bleckwedel et al. [74] found differences in promoter sequences for each gene and suggested a differential expression regulated by specific environmental conditions; nonetheless, the quantification of D-LDH transcripts is needed to confirm this hypothesis.

Regarding other genes related with the central metabolism, a gene involved in the use of oxygen for NAD(P)H reoxidation (*yjlD*) was strictly present in clade 2 only. NADH dehydrogenases are a key component of the respiratory chain, but no other gene used for the quinone pool was found in *Fructobacillus* ([38] and this study), suggesting that *yjlD* is involved in the oxidation of NAD(P)H under the presence of oxygen. In addition, genes for the synthesis of exopolysaccharides (EPS) were widely spread among the studied strains. Genes encoding putative levansucrases and other fructosyltransferases were found in several *Fructobacillus* genomes, as previously observed by Endo et al. [38]. In addition, the detection of genes with domains for glycosyltransferases was reported in this work for the first time for *Fructobacillus* spp. Although Endo et al. [38] failed to detect EPS production in some *Fructobacillus* type strains, Tahir et al. [75] found two EPS-producer *F*. *fructosus* strains able to synthesize levan and dextran.

Optimization of mannitol production by bacteria is of high interest since its technological relevance in food industry [76]. It is known that *Fructobacillus* organisms are efficient mannitol producers [29] due to its requirement of fructose as electron acceptor [25]. The genes responsible for the mannitol synthesis and its entrance into the cell (*mdh* and *fruP*, respectively) were present in all analyzed genomes, indicating the importance of these genes for the fructophilic metabolism in *Fructobacillus*. However, FK and GPI enzymes can compete against MDH for fructose by phosphorylating this sugar and channeling it into the phosphoketolase pathway, respectively. Affinity of fructokinases for fructose is higher than that of mannitol dehydrogenase, this being a hurdle in mannitol production [77, 78]. In this work, remarkable differences were detected in *fk* and *gpi* genes among the analyzed genomes. It would be of high relevance to deeply characterize these differences in *fk* and *gpi* genes and its regulation mechanisms. Helanto et al. [79] improved the yield of mannitol from fructose by inactivation of the *fk* gene in *L*. *pseudomesenteroides*. These authors also observed a higher rate of fructose consumption when *fk* was inactivated. According to these results, a low FK activity would be desirable to enhance mannitol biosynthesis and guarantee a rapid decrease of the fructose content in food fermentation. Surprisingly, the deletion of the FK activity was not totally achieved in the *fk* mutant designed by [79], despite no *fk* transcript being detected in this strain. A possible reason could implicate other uncharacterized enzymes with FK activity. This hypothesis could explain the absence of an *fk* gene in *F*. *broussonetiae* M2-14^T^ reported in the present work, and the ability of this strain to grow in fructose as a sole carbon source [11]. Further studies related to the downregulation of FK and GPI activity will allow exploiting the use of these bacteria as mannitol producers in the fermentation of fructose-rich matrices.

## Conclusions

A comparative genomic analysis of the currently available *Fructobacillus* genomes was performed. The results of this study allowed us to distinguish two phylogenetic groups, where the organisms of clade 1 showed a simplified machinery for the biosynthesis of nitrogen compounds. Furthermore, differences were also identified in the presence of mobile elements and in genes with an essential function in the use of fructose and electron acceptors among the studied strains, being these differences clade-independent. These findings contribute to a better understanding of the unusual metabolism of these organisms that may be exploited for future biotechnological applications.

## Supporting information

S1 Table Main characteristics of prophages found in *Fructobacillus* genomes. (DOCX)

## References

[1] Endo A, Okada S. Reclassification of the genus *Leuconostoc* and proposals of *Fructobacillus fructosus* gen. nov., comb. nov., *Fructobacillus durionis* comb. nov., *Fructobacillus ficulneus* comb. nov. and *Fructobacillus pseudoficulneus* comb. nov. Int J Syst Evol Microbiol. 2008;58(Pt 9):2195–205. Epub 2008/09/05. doi: 10.1099/ijs.0.65609–0 . 18768629

[2] Endo A, Tanaka N, Oikawa Y, Okada S, Dicks L. Fructophilic characteristics of *Fructobacillus* spp. may be due to the absence of an alcohol/acetaldehyde dehydrogenase gene (*adhE*). Curr Microbiol. 2014;68(4):531–5. Epub 2013/12/20. doi: 10.1007/s00284-013-0506-3 . 24352296

[3] Zheng J, Wittouck S, Salvetti E, Franz C, Harris HMB, Mattarelli P, et al. A taxonomic note on the genus *Lactobacillus*: Description of 23 novel genera, emended description of the genus *Lactobacillus* Beijerinck 1901, and union of *Lactobacillaceae* and *Leuconostocaceae*. Int J Syst Evol Microbiol. 2020;70(4):2782–858. Epub 2020/04/16. doi: 10.1099/ijsem.0.004107 . 32293557

[4] Endo A, Dicks LMT. The genus *Fructobacillus*. Lactic Acid Bacteria 2014. p. 381–9.

[5] Maeno S, Kajikawa A, Dicks L, Endo A. Introduction of bifunctional alcohol/acetaldehyde dehydrogenase gene (*adhE*) in *Fructobacillus fructosus* settled its fructophilic characteristics. Res Microbiol. 2019;170(1):35–42. Epub 2018/10/07. doi: 10.1016/j.resmic.2018.09.004 . 30291951

[6] Endo A, Futagawa-Endo Y, Dicks LM. Isolation and characterization of fructophilic lactic acid bacteria from fructose-rich niches. Syst Appl Microbiol. 2009;32(8):593–600. Epub 2009/09/08. doi: 10.1016/j.syapm.2009.08.002 . 19733991

[7] Endo A. Fructophilic lactic acid bacteria inhabit fructose-rich niches in nature. Microb Ecol Health Dis. 2012;23(1):18563. Epub 2012/01/01. doi: 10.3402/mehd.v23i0.18563 ; PubMed Central PMCID: PMC3747758. 23990834 PMC3747758

[8] Endo A, Salminen S. Honeybees and beehives are rich sources for fructophilic lactic acid bacteria. Syst Appl Microbiol. 2013;36(6):444–8. Epub 2013/07/13. doi: 10.1016/j.syapm.2013.06.002 . 23845309

[9] He H, Chen Y, Zhang Y, Wei C. Bacteria associated with gut lumen of *Camponotus japonicus* Mayr. Environ Entomol. 2011;40(6):1405–9. doi: 10.1603/en11157 22217755

[10] Koch H, Schmid-Hempel P. Bacterial communities in central european bumblebees: low diversity and high specificity. Microb Ecol. 2011;62(1):121–33. doi: 10.1007/s00248-011-9854-3 21556885

[11] Lin S-T, Guu J-R, Wang H-M, Tamura T, Mori K, Huang L, et al. *Fructobacillus papyriferae* sp. nov., *Fructobacillus papyrifericola* sp. nov., *Fructobacillus broussonetiae* sp. nov. and *Fructobacillus parabroussonetiae* sp. nov., isolated from paper mulberry in Taiwan. Int J Syst Evol Microbiol. 2022;72(2):005235. doi: 10.1099/ijsem.0.005235 35138243

[12] Rokop ZP, Horton MA, Newton IL. Interactions between cooccurring lactic acid bacteria in honey bee hives. Appl Environ Microbiol. 2015;81(20):7261–70. Epub 2015/08/09. doi: 10.1128/AEM.01259-15 ; PubMed Central PMCID: PMC4579437. 26253685 PMC4579437

[13] Snauwaert I, Papalexandratou Z, De Vuyst L, Vandamme P. Characterization of strains of *Weissella fabalis* sp. nov. and *Fructobacillus tropaeoli* from spontaneous cocoa bean fermentations. Int J Syst Evol Microbiol. 2013;63(Pt 5):1709–16. Epub 2012/08/28. doi: 10.1099/ijs.0.040311–0 . 22922535

[14] Thaochan N, Drew RA, Hughes JM, Vijaysegaran S, Chinajariyawong A. Alimentary tract bacteria isolated and identified with API-20E and molecular cloning techniques from Australian tropical fruit flies, *Bactrocera cacuminata* and *B*. *tryoni*. J Insect Sci. 2010;10:131. Epub 2010/10/05. doi: 10.1673/031.010.13101 ; PubMed Central PMCID: PMC3016917. 20883132 PMC3016917

[15] Filannino P, Di Cagno R, Addante R, Pontonio E, Gobbetti M. Metabolism of fructophilic lactic acid bacteria isolated from the *Apis mellifera* L. Bee Gut: phenolic acids as external electron acceptors. Appl Environ Microbiol. 2016;82(23):6899–911. Epub 2016/09/18. doi: 10.1128/AEM.02194-16 ; PubMed Central PMCID: PMC5103089. 27637884 PMC5103089

[16] Pachla A, Wicha M, Ptaszynska AA, Borsuk G, Trokenheim LL, Malek W. The molecular and phenotypic characterization of fructophilic lactic acid bacteria isolated from the guts of *Apis mellifera* L. derived from a Polish apiary. J Appl Genet. 2018;59(4):503–14. Epub 2018/10/01. doi: 10.1007/s13353-018-0467-0 . 30269313

[17] Praet J, Meeus I, Cnockaert M, Houf K, Smagghe G, Vandamme P. Novel lactic acid bacteria isolated from the bumble bee gut: *Convivina intestini* gen. nov., sp. nov., *Lactobacillus bombicola* sp. nov., and *Weissella bombi* sp. nov. Antonie Van Leeuwenhoek. 2015;107(5):1337–49. Epub 2015/03/19. doi: 10.1007/s10482-015-0429-z . 25783976

[18] Janashia I, Alaux C. Specific immune stimulation by endogenous bacteria in honey bees (Hymenoptera: Apidae). J Econ Entomol. 2016;109(3):1474–7. Epub 2016/04/12. doi: 10.1093/jee/tow065 . 27063842

[19] Chuah LO, Shamila-Syuhada AK, Liong MT, Rosma A, Thong KL, Rusul G. Physio-chemical, microbiological properties of tempoyak and molecular characterisation of lactic acid bacteria isolated from tempoyak. Food Microbiol. 2016;58:95–104. Epub 2016/05/25. doi: 10.1016/j.fm.2016.04.002 . 27217364

[20] Papalexandratou Z, Falony G, Romanens E, Jimenez JC, Amores F, Daniel HM, et al. Species diversity, community dynamics, and metabolite kinetics of the microbiota associated with traditional ecuadorian spontaneous cocoa bean fermentations. Appl Environ Microbiol. 2011;77(21):7698–714. Epub 2011/09/20. doi: 10.1128/AEM.05523-11 ; PubMed Central PMCID: PMC3209185. 21926224 PMC3209185

[21] Viesser JA, de Melo Pereira GV, de Carvalho Neto DP, Vandenberghe LPdS, Azevedo V, Brenig B, et al. Exploring the contribution of fructophilic lactic acid bacteria to cocoa beans fermentation: isolation, selection and evaluation. Food Res Int. 2020;136:109478. doi: 10.1016/j.foodres.2020.109478 32846561

[22] Filannino P, Di Cagno R, Tlais AZA, Cantatore V, Gobbetti M. Fructose-rich niches traced the evolution of lactic acid bacteria toward fructophilic species. Crit Rev Microbiol. 2019;45(1):65–81. Epub 2019/01/22. doi: 10.1080/1040841X.2018.1543649 . 30663917

[23] Mai BH, Yan L-J. The negative and detrimental effects of high fructose on the liver, with special reference to metabolic disorders. Diabet Metab Synd Ob. 2019;12:821. doi: 10.2147/DMSO.S198968 31213868 PMC6549781

[24] Tappy L, Lê K-A. Metabolic effects of fructose and the worldwide increase in obesity. Physiol Rev. 2010. doi: 10.1152/physrev.00019.2009 20086073

[25] Endo A, Maeno S, Tanizawa Y, Kneifel W, Arita M, Dicks L, et al. Fructophilic lactic acid bacteria, a unique group of fructose-fermenting microbes. Appl Environ Microbiol. 2018;84(19):e01290–18. Epub 2018/07/29. doi: 10.1128/AEM.01290-18 ; PubMed Central PMCID: PMC6146980. 30054367 PMC6146980

[26] Chen M, Zhang W, Wu H, Guang C, Mu W. Mannitol: physiological functionalities, determination methods, biotechnological production, and applications. Appl Microbiol Biotechnol. 2020;104(16):6941–51. Epub 2020/07/01. doi: 10.1007/s00253-020-10757-y . 32601737

[27] Saha BC, Racine FM. Biotechnological production of mannitol and its applications. Appl Microbiol Biotechnol. 2011;89(4):879–91. Epub 2010/11/11. doi: 10.1007/s00253-010-2979-3 . 21063702

[28] Behare PV, Mazhar S, Pennone V, McAuliffe O. Evaluation of lactic acid bacteria strains isolated from fructose-rich environments for their mannitol-production and milk-gelation abilities. J Dairy Sci. 2020;103(12):11138–51. doi: 10.3168/jds.2020-19120 33010917

[29] Ruiz Rodriguez LG, Aller K, Bru E, De Vuyst L, Hebert EM, Mozzi F. Enhanced mannitol biosynthesis by the fruit origin strain *Fructobacillus tropaeoli* CRL 2034. Appl Microbiol Biotechnol. 2017;101(15):6165–77. Epub 2017/07/05. doi: 10.1007/s00253-017-8395-1 . 28674850

[30] Ruiz Rodriguez LG, Mohamed F, Bleckwedel J, Medina R, De Vuyst L, Hebert EM, et al. Diversity and functional properties of lactic acid bacteria isolated from wild fruits and flowers present in northern argentina. Front Microbiol. 2019;10:1091. Epub 2019/06/06. doi: 10.3389/fmicb.2019.01091 ; PubMed Central PMCID: PMC6536596. 31164879 PMC6536596

[31] Saha BC, Nakamura LK. Production of mannitol and lactic acid by fermentation with *Lactobacillus intermedius* NRRL B-3693. Biotechnol Bioeng. 2003;82(7):864–71. Epub 2003/04/18. doi: 10.1002/bit.10638 . 12701154

[32] Yun JW, Kim DH. A comparative study of mannitol production by two lactic acid bacteria. J Fermentation Bioeng. 1998;85(2):203–8.

[33] Al-Nabulsi AA, Olaimat AN, Osaili TM, Shaker RR, Elabedeen NZ, Jaradat ZW, et al. Use of acetic and citric acids to control *Salmonella Typhimurium* in tahini (sesame paste). Food Microbiol. 2014;42:102–8. 24929724 10.1016/j.fm.2014.02.020

[34] Dicks L, Endo A. Are fructophilic lactic acid bacteria (FLAB) beneficial to humans? Benef Microbes. 2022;13(1):3–11. doi: 10.3920/BM2021.0044 35144525

[35] Garcia M, Amalaradjou MAR, Nair MKM, Annamalai T, Surendranath S, Lee S, et al. Inactivation of *Listeria monocytogenes* on frankfurters by monocaprylin alone or in combination with acetic acid. J Food Prot. 2007;70(7):1594–9. 17685330 10.4315/0362-028x-70.7.1594

[36] Perez-Diaz IM, Hayes J, Medina E, Anekella K, Daughtry K, Dieck S, et al. Reassessment of the succession of lactic acid bacteria in commercial cucumber fermentations and physiological and genomic features associated with their dominance. Food Microbiol. 2017;63:217–27. Epub 2017/01/04. doi: 10.1016/j.fm.2016.11.025 . 28040172

[37] Acin Albiac M, Di Cagno R, Filannino P, Cantatore V, Gobbetti M. How fructophilic lactic acid bacteria may reduce the FODMAPs content in wheat-derived baked goods: a proof of concept. Microb Cell Factories. 2020;19(1):182. Epub 2020/09/19. doi: 10.1186/s12934-020-01438-6 ; PubMed Central PMCID: PMC7499921. 32943064 PMC7499921

[38] Endo A, Tanizawa Y, Tanaka N, Maeno S, Kumar H, Shiwa Y, et al. Comparative genomics of *Fructobacillus* spp. and *Leuconostoc* spp. reveals niche-specific evolution of *Fructobacillus* spp. BMC Genom. 2015;16(1):1–13. Epub 2015/12/31. doi: 10.1186/s12864-015-2339-x ; PubMed Central PMCID: PMC4696137. 26715526 PMC4696137

[39] Endo A, Futagawa-Endo Y, Sakamoto M, Kitahara M, Dicks LMT. *Lactobacillus florum* sp. nov., a fructophilic species isolated from flowers. Int J Syst Evol Microbiol. 2010;60(10):2478–82. Epub 2009/12/08. doi: 10.1099/ijs.0.019067–0 . 19965998

[40] Maeno S, Dicks L, Nakagawa J, Endo A. *Lactobacillus apinorum* belongs to the fructophilic lactic acid bacteria. Biosci Microbiota Food Health. 2017:17–008. doi: 10.12938/bmfh.17-008 29038770 PMC5633529

[41] Maeno S, Tanizawa Y, Kanesaki Y, Kubota E, Kumar H, Dicks L, et al. Genomic characterization of a fructophilic bee symbiont *Lactobacillus kunkeei* reveals its niche-specific adaptation. Syst Appl Microbiol. 2016;39(8):516–26. Epub 2016/10/26. doi: 10.1016/j.syapm.2016.09.006 . 27776911

[42] Ruiz Rodriguez LG, Mohamed F, Bleckwedel J, Teran LC, Hebert EM, Mozzi F, et al. Exploring the genome of *Fructobacillus tropaeoli* CRL 2034, a fig-origin strain that produces high levels of mannitol from fructose. Curr Microbiol. 2020;77(9):2215–25. Epub 2020/07/01. doi: 10.1007/s00284-020-02102-3 . 32601836

[43] Bolger AM, Lohse M, Usadel B. Trimmomatic: a flexible trimmer for Illumina sequence data. Bioinformatics. 2014;30(15):2114–20. doi: 10.1093/bioinformatics/btu170 24695404 PMC4103590

[44] Bankevich A, Nurk S, Antipov D, Gurevich AA, Dvorkin M, Kulikov AS, et al. SPAdes: a new genome assembly algorithm and its applications to single-cell sequencing. J Comput Biol. 2012;19(5):455–77. doi: 10.1089/cmb.2012.0021 22506599 PMC3342519

[45] Gurevich A, Saveliev V, Vyahhi N, Tesler G. QUAST: quality assessment tool for genome assemblies. Bioinformatics. 2013;29(8):1072–5. doi: 10.1093/bioinformatics/btt086 23422339 PMC3624806

[46] Parks DH, Imelfort M, Skennerton CT, Hugenholtz P, Tyson GW. CheckM: assessing the quality of microbial genomes recovered from isolates, single cells, and metagenomes. Genome Res. 2015;25(7):1043–55. Epub 2015/05/16. doi: 10.1101/gr.186072.114 ; PubMed Central PMCID: PMC4484387. 25977477 PMC4484387

[47] Couvin D, Bernheim A, Toffano-Nioche C, Touchon M, Michalik J, Néron B, et al. CRISPRCasFinder, an update of CRISPRFinder, includes a portable version, enhanced performance and integrates search for Cas proteins. Nucleic Acids Res. 2018;46(W1):W246–W51. doi: 10.1093/nar/gky425 29790974 PMC6030898

[48] Arndt D, Grant JR, Marcu A, Sajed T, Pon A, Liang Y, et al. PHASTER: a better, faster version of the PHAST phage search tool. Nucleic Acids Res. 2016;44(W1):W16–W21. doi: 10.1093/nar/gkw387 27141966 PMC4987931

[49] Siguier P, Pérochon J, Lestrade L, Mahillon J, Chandler M. ISfinder: the reference centre for bacterial insertion sequences. Nucleic Acids Res. 2006;34(suppl_1):D32–D6. doi: 10.1093/nar/gkj014 16381877 PMC1347377

[50] van Heel AJ, de Jong A, Song C, Viel JH, Kok J, Kuipers OP. BAGEL4: a user-friendly web server to thoroughly mine RiPPs and bacteriocins. Nucleic Acids Res. 2018;46(W1):W278–W81. doi: 10.1093/nar/gky383 29788290 PMC6030817

[51] Gupta SK, Padmanabhan BR, Diene SM, Lopez-Rojas R, Kempf M, Landraud L, et al. ARG-ANNOT, a new bioinformatic tool to discover antibiotic resistance genes in bacterial genomes. Antimicrob Agents Chemother. 2014;58(1):212–20. doi: 10.1128/AAC.01310-13 24145532 PMC3910750

[52] Bortolaia V, Kaas RS, Ruppe E, Roberts MC, Schwarz S, Cattoir V, et al. ResFinder 4.0 for predictions of phenotypes from genotypes. J Antimicrob Chemother. 2020;75(12):3491–500. doi: 10.1093/jac/dkaa345 32780112 PMC7662176

[53] Seemann T. Prokka: rapid prokaryotic genome annotation. Bioinformatics. 2014;30(14):2068–9. doi: 10.1093/bioinformatics/btu153 24642063

[54] Kanehisa M, Sato Y, Morishima K. BlastKOALA and GhostKOALA: KEGG tools for functional characterization of genome and metagenome sequences. J Mol Biol. 2016;428(4):726–31. doi: 10.1016/j.jmb.2015.11.006 26585406

[55] Cantalapiedra CP, Hernández-Plaza A, Letunic I, Bork P, Huerta-Cepas J. eggNOG-mapper v2: Functional Annotation, Orthology Assignments, and Domain Prediction at the Metagenomic Scale. Mol Biol Evol. 2021;38(12):5825–9. doi: 10.1093/molbev/msab293 34597405 PMC8662613

[56] Zhang H, Yohe T, Huang L, Entwistle S, Wu P, Yang Z, et al. dbCAN2: a meta server for automated carbohydrate-active enzyme annotation. Nucleic Acids Res. 2018;46(W1):W95–W101. doi: 10.1093/nar/gky418 29771380 PMC6031026

[57] Nguyen L-T, Schmidt HA, von Haeseler A, Minh BQ. IQ-TREE: A fast and effective stochastic algorithm for estimating maximum-likelihood phylogenies. Mol Biol Evol. 2014;32(1):268–74. doi: 10.1093/molbev/msu300 25371430 PMC4271533

[58] Sievers F, Higgins DG. Clustal Omega, accurate alignment of very large numbers of sequences. In: Russell D, editor. Multiple Sequence Alignment Methods Methods in Molecular Biology. 1079. Totowa, NJ: Humana Press; 2014. p. 105–16. 10.1007/978-1-62703-646-7_6 24170397

[59] Talavera G, Castresana J. Improvement of phylogenies after removing divergent and ambiguously aligned blocks from protein sequence alignments. Syst Biol. 2007;56(4):564–77. doi: 10.1080/10635150701472164 17654362

[60] Didelot X, Wilson DJ. ClonalFrameML: efficient inference of recombination in whole bacterial genomes. PLoS Comput Biol. 2015;11(2):e1004041. Epub 2015/02/13. doi: 10.1371/journal.pcbi.1004041 ; PubMed Central PMCID: PMC4326465. 25675341 PMC4326465

[61] Contreras-Moreira B, Vinuesa P. GET_HOMOLOGUES, a versatile software package for scalable and robust microbial pangenome analysis. Appl Environ Microbiol. 2013;79(24):7696–701. doi: 10.1128/AEM.02411-13 24096415 PMC3837814

[62] Li L, Stoeckert CJ Jr., Roos DS. OrthoMCL: identification of ortholog groups for eukaryotic genomes. Genome Res. 2003;13(9):2178–89. Epub 2003/09/04. doi: 10.1101/gr.1224503 ; PubMed Central PMCID: PMC403725. 12952885 PMC403725

[63] Deboutte W, Beller L, Yinda CK, Maes P, de Graaf DC, Matthijnssens J. Honey-bee-associated prokaryotic viral communities reveal wide viral diversity and a profound metabolic coding potential. Proc. Natl. Acad. Sci. U.S.A. 2020;117(19):10511–9. Epub 2020/04/29. doi: 10.1073/pnas.1921859117 ; PubMed Central PMCID: PMC7229680. 32341166 PMC7229680

[64] Siguier P, Filée J, Chandler M. Insertion sequences in prokaryotic genomes. Curr Opin Microbiol. 2006;9(5):526–31. doi: 10.1016/j.mib.2006.08.005 16935554

[65] Yarza P, Yilmaz P, Pruesse E, Glockner FO, Ludwig W, Schleifer KH, et al. Uniting the classification of cultured and uncultured bacteria and archaea using 16S rRNA gene sequences. Nat Rev Microbiol. 2014;12(9):635–45. Epub 2014/08/15. doi: 10.1038/nrmicro3330 . 25118885

[66] Batut B, Knibbe C, Marais G, Daubin V. Reductive genome evolution at both ends of the bacterial population size spectrum. Nat Rev Microbiol. 2014;12(12):841–50. Epub 2014/09/16. doi: 10.1038/nrmicro3331 . 25220308

[67] Bourguignon T, Kinjo Y, Villa-Martin P, Coleman NV, Tang Q, Arab DA, et al. Increased Mutation Rate Is Linked to Genome Reduction in Prokaryotes. Curr Biol. 2020;30(19):3848–55 e4. Epub 2020/08/09. doi: 10.1016/j.cub.2020.07.034 . 32763167

[68] Tamarit D, Ellegaard KM, Wikander J, Olofsson T, Vasquez A, Andersson SG. Functionally structured genomes in *Lactobacillus kunkeei* colonizing the honey crop and food products of honeybees and stingless bees. Genome Biol Evol. 2015;7(6):1455–73. Epub 2015/05/09. doi: 10.1093/gbe/evv079 ; PubMed Central PMCID: PMC4494060. 25953738 PMC4494060

[69] Kuo C-H, Ochman H. Deletional bias across the three domains of life. Genome Biol Evol. 2009;1:145–52. doi: 10.1093/gbe/evp016 20333185 PMC2817411

[70] Mira A, Ochman H, Moran NA. Deletional bias and the evolution of bacterial genomes. Trends Genet. 2001;17(10):589–96. doi: 10.1016/s0168-9525(01)02447-7 11585665

[71] Mandrioli R, Mercolini L, Raggi MA. Recent trends in the analysis of amino acids in fruits and derived foodstuffs. Anal Bioanal Chem. 2013;405(25):7941–56. Epub 2013/05/21. doi: 10.1007/s00216-013-7025-8 . 23686004

[72] Zheng H, Perreau J, Powell JE, Han B, Zhang Z, Kwong WK, et al. Division of labor in honey bee gut microbiota for plant polysaccharide digestion. Proc. Natl. Acad. Sci. U.S.A 2019;116(51):25909–16. Epub 2019/11/30. doi: 10.1073/pnas.1916224116 ; PubMed Central PMCID: PMC6926048. 31776248 PMC6926048

[73] Maeno S, Nishimura H, Tanizawa Y, Dicks L, Arita M, Endo A. Unique niche-specific adaptation of fructophilic lactic acid bacteria and proposal of three *Apilactobacillus* species as novel members of the group. BMC Microbiol. 2021;21(1):1–14. Epub 2021/02/11. doi: 10.1186/s12866-021-02101-9 ; PubMed Central PMCID: PMC7871557. 33563209 PMC7871557

[74] Bleckwedel J, Mohamed F, Mozzi F, Raya RR. Major role of lactate dehydrogenase D-LDH1 for the synthesis of lactic acid in *Fructobacillus tropaeoli* CRL 2034. Appl Microbiol Biotechnol. 2020;104(17):7409–26. Epub 2020/07/16. doi: 10.1007/s00253-020-10776-9 . 32666186

[75] Tahir M, Majeed MI, Nawaz H, Ali S, Rashid N, Kashif M, et al. Raman spectroscopy for the analysis of different exo-polysaccharides produced by bacteria. Spectrochim. Acta A Mol. Biomol. Spectrosc. 2020;237:118408. Epub 2020/05/07. doi: 10.1016/j.saa.2020.118408 . 32371352

[76] Patra F, Tomar SK, Arora S. Technological and functional applications of low-calorie sweeteners from lactic acid bacteria. J Food Sci. 2009;74(1):R16–23. Epub 2009/02/10. doi: 10.1111/j.1750-3841.2008.01005.x . 19200114

[77] von Weymarn N. Process Development for Mannitol Production by Lactid Acid Bacteria: Helsinki University of Technology; 2002.

[78] Wisselink H, Weusthuis R, Eggink G, Hugenholtz J, Grobben G. Mannitol production by lactic acid bacteria: a review. Int Dairy J. 2002;12(2–3):151–61.

[79] Helanto M, Aarnikunnas J, von Weymarn N, Airaksinen U, Palva A, Leisola M. Improved mannitol production by a random mutant of *Leuconostoc pseudomesenteroides*. J Biotechnol. 2005;116(3):283–94. Epub 2005/02/15. doi: 10.1016/j.jbiotec.2004.11.001 . 15707689

