# Supplementary material for: Genomic diversity in Fructobacillus spp. isolated from fructose-rich niches
Source: PLoS One. 2023 Feb 16;18(2):e0281839. doi: 10.1371/journal.pone.0281839 (PMC9934391; doi:10.1371/journal.pone.0281839)
Supplement: S1 Table — (DOCX) [file pone.0281839.s001.docx]

**S1 Table. Main characteristics of prophages found in *Fructobacillus* genomes**

|  | Length | Score* | tRNA | Phage proteins | Hypothetical proteins | Total no. of proteins | Attachment site | % GC | BLASTn best hit** | % identity best hit** | % coverage best hit** |
| --- | --- | --- | --- | --- | --- | --- | --- | --- | --- | --- | --- |
| *F. sp.* CRL 2054 (1) | 31.8 Kb | Intact (150) | No | 31 | 20 | 51 | Yes | 41.10% | Bacteriophage sp. 103 (*Apis mellifera*) | 67.00% | 14.00% |
| *F. sp*. CRL 2054 (2) | 31.6 Kb | Intact (120) | No | 26 | 21 | 47 | Yes | 41.89% | Bacteriophage sp. 103 (*Apis mellifera*) | 68.00% | 12.00% |
| *F. fructosus* KCTC 3544^T^ | 16 Kb | Questionable (90) | No | 17 | 7 | 24 | No | 41.03% | *Siphoviridae* sp. 414 (*Apis mellifera*) | 87.00% | 82.00% |
| *F. fructosus* DPC 7238 | 24.3 Kb | Intact (120) | No | 22 | 7 | 29 | No | 41.72% | Bacteriophage sp. Isolate ct68T10 | 68.00% | 27.00% |
| *F. fructosus* strain 13 | 18.5 Kb | Questionable (90) | No | 21 | 4 | 25 | No | 40.44% | *Siphoviridae* sp. 414 (*Apis mellifera*) | 88.00% | 85.00% |
| *F. fructosus* MAG1 | 36.6 Kb | Intact (130) | No | 37 | 22 | 59 | Yes | 39.79% | *Siphoviridae* sp. 414 (*Apis mellifera*) | 82.64% | 37.00% |
| *F. fructosus* MAG2 (1) | 33.1 Kb | Intact (100) | No | 33 | 16 | 49 | No | 40.05% | *Siphoviridae* sp. 414 (*Apis mellifera*) | 88.40% | 49.00% |
| *F. fructosus* MAG2 (2) | 24.8 Kb | Intact (140) | No | 24 | 14 | 38 | No | 42.44% | Bacteriophage sp. ct68T10 (Human metagenome) | 68.82% | 26.00% |
| *F. papyriferae* M1-10 | 54.9 Kb | Intact (150) | Yes (Cys-tRNA) | 43 | 36 | 79 | Yes | 41.86% | *Siphoviridae* sp. 414 (*Apis mellifera*) | 84.00% | 36.00% |
| *F. papyriferae* M1-13 | 54.9 Kb | Intact (150) | Yes (Cys-tRNA) | 43 | 34 | 77 | Yes | 41.86% | *Siphoviridae* sp. 414 (*Apis mellifera*) | 84.00% | 36.00% |
| *F. papyrifericola* M1-21 | 44.4 Kb | Intact (120) | No | 42 | 27 | 69 | Yes | 40.58% | *Siphoviridae* sp. 414 (*Apis mellifera*) | 86.00% | 40.00% |
| *F.* sp M158 (1) | 18.5 Kb | Intact (140) | No | 18 | 7 | 25 | No | 43.40% | *Siphoviridae* sp. ct6md14 (Human metagenome) | 67.56% | 32.00% |
| *F. sp* M158 (2) | 17.2 Kb | Questionable (70) | No | 13 | 7 | 20 | Yes | 38.94% | Bacteriophage bIL309  (*Lactococcus* phage) | 64.37% | 12.00% |
| *F. sp* M158 (3) | 12.6 Kb | Questionable (70) | No | 6 | 7 | 13 | Yes | 34.00% | *Siphoviridae* sp. 29 (*Apis mellifera*) | 68.51% | 6.00% |
| *F. apis* W13 | 31.0 Kb | Intact (110) | No | 27 | 14 | 41 | No | 40.57% | *Siphoviridae* sp. 414 (*Apis mellifera*) | 83.00% | 52.00% |
| *F. ficulneus* JCM 12225^T^ | 30.5 Kb | Intact (100) | Yes (Asn-tRNA) | 33 | 22 | 55 | No | 39.80% | *Siphoviridae* sp. 414 (*Apis mellifera*) | 71.00% | 60.00% |
| *F.* sp. EFB-N1 (1) | 22.5 Kb | Intact (150) | No | 23 | 10 | 33 | No | 40.72% | *Siphoviridae* sp. 414 (*Apis mellifera*) | 90.00% | 67.00% |
| *F.* sp. EFB-N1 (2) | 16.6 Kb | Questionable (80) | Yes (Leu-tRNA) | 17 | 9 | 26 | No | 39.51% | Bacteriophage sp. ct68T10 (Human metagenome) | 68.00% | 32.00% |
| *F. tropaeoli* F214-1^T^ (1) | 23.8 Kb | Intact (120) | No | 24 | 8 | 32 | No | 42.05% | *Myoviridae* sp. 302 (*Apis mellifera*) | 95.00% | 9.00% |
| *F. tropaeoli* F214-1^T^ (2) | 20.6 Kb | Intact (100) | No | 23 | 4 | 27 | No | 40.92% | *Siphoviridae* sp. 414 (*Apis mellifera*) | 90.00% | 79.00% |
| *F. tropaeoli* CRL 2034 (1) | 32.8 Kb | Questionable (70) | No | 32 | 18 | 50 | Yes | 40.28% | *Myoviridae* sp. 302 (*Apis mellifera*) | 88.00% | 14.00% |
| *F. tropaeoli* CRL 2034 (2) | 36.1 Kb | Intact (120) | Yes (Asn-tRNA) | 34 | 28 | 62 | Yes | 38.84% | *Siphoviridae* sp. 414 (*Apis mellifera*) | 83.00% | 58.00% |
| *F. tropaeoli* RD012353 | 40.1 Kb | Intact (150) | Yes (Arg-tRNA) | 41 | 22 | 63 | Yes | 40.34% | Bacteriophage sp. ct68T10 (Human metagenome) | 68.00% | 19.00% |
| *F. cardui*  M131 (1) (contig 1; nt 2578-42485) | 39.9 Kb | Intact (100) | No | 23 | 24 | 47 | Yes | 41.39% | Bacteriophage sp. ct68T10 (Human metagenome) | 68.26% | 15.00% |
| *F. cardui*  M131 (2) (contig 3; nt 125-30606) | 30.4 Kb | Questionable (80) | No | 26 | 17 | 43 | No | 40.47% | *Myoviridae* sp. 302 (*Apis mellifera*) | 93.67% | 81.00% |
| *F. cardui*  M131 (3) (contig 16; nt 98-18030) | 17.9 Kb | Questionable (80) | No | 18 | 5 | 23 | No | 41.10% | *Siphoviridae* sp. 414 (*Apis mellifera*) | 89.05% | 78.00% |
| *F.* sp. KI3_B9 (nt 1376305-1412524) | 36.2 Kb | Intact (120) | No | 26 | 21 | 47 | Yes | 40.99% | *Siphoviridae* sp. ct6md14 (Human metagenome) | 69.20% | 13.00% |
| *F.* sp. MAG4 (cont 1; nt 17769-33253) | 15.4 Kb | Questionable (70) | No | 12 | 5 | 17 | Yes | 37.50% | Bacteriophage bIL309 (*Lactococcus* phage) | 64.55% | 13.00% |

*Assigned score through PHASTER tool (Intact: 90 – 150, Questionable: 70 – 90)

**Best result of the BLASTn search against sequences of viruses and its corresponding identity and coverage percentages.
